# Developmental Modulation of Root Cell Wall Architecture Confers Resistance to an Oomycete Pathogen

**DOI:** 10.1016/j.cub.2020.08.011

**Published:** 2020-11-02

**Authors:** Aleksandr Gavrin, Thomas Rey, Thomas A. Torode, Justine Toulotte, Abhishek Chatterjee, Jonathan Louis Kaplan, Edouard Evangelisti, Hiroki Takagi, Varodom Charoensawan, David Rengel, Etienne-Pascal Journet, Frédéric Debellé, Fernanda de Carvalho-Niebel, Ryohei Terauchi, Siobhan Braybrook, Sebastian Schornack

**Affiliations:** 1 Sainsbury Laboratory (SLCU), University of Cambridge, 47 Bateman Street, Cambridge CB2 1LR, UK; 2 Iwate Biotechnology Institute, 22-174-4 Narita, Kitakami, Iwate 024-0003, Japan; 3 LIPM, Université de Toulouse, INRA, CNRS, Castanet-Tolosan 31326, France; 4 Department of Molecular, Cell, and Developmental Biology, 610 Charles E Young Drive South, University of California, Los Angeles, Los Angeles, CA 90095, USA; 5 Molecular Biology Institute, University of California, Los Angeles, Los Angeles, CA 90095, USA; 6 Eli and Edythe Broad Center of Regenerative Medicine and Stem Cell Research, University of California, Los Angeles, Los Angeles, CA 90095, USA; 7 Department of Biochemistry, Faculty of Science, and Integrative Computational BioScience (ICBS) Center, Mahidol University, Bangkok 10400, Thailand; 8 GeT-PlaGe, Genotoul, INRA US1426, Castanet-Tolosan Cedex, France; 9 AGIR, Université de Toulouse, INRA, ENSFEA, Castanet-Tolosan 31326, France

**Keywords:** susceptibility gene, SCAR/WAVE, *Phytophthora*, disease resistance, cell wall, root endosymbiosis, Rhizobia, *Medicago truncatula*

## Abstract

The cell wall is the primary interface between plant cells and their immediate environment and must balance multiple functionalities, including the regulation of growth, the entry of beneficial microbes, and protection against pathogens. Here, we demonstrate how API, a SCAR2 protein component of the SCAR/WAVE complex, controls the root cell wall architecture important for pathogenic oomycete and symbiotic bacterial interactions in legumes. A mutation in *API* results in root resistance to the pathogen *Phytophthora palmivora* and colonization defects by symbiotic rhizobia. Although *api* mutant plants do not exhibit significant overall growth and development defects, their root cells display delayed actin and endomembrane trafficking dynamics and selectively secrete less of the cell wall polysaccharide xyloglucan. Changes associated with a loss of *API* establish a cell wall architecture with altered biochemical properties that hinder *P. palmivora* infection progress. Thus, developmental stage-dependent modifications of the cell wall, driven by SCAR/WAVE, are important in balancing cell wall developmental functions and microbial invasion.

## Highlights

• The SCAR protein API controls actin and endomembrane trafficking dynamics
• SCAR proteins of several plant species can support symbiosis and pathogen infection
• A mutation in *API* affects specific biochemical properties of plant cell walls
• An altered wall architecture results in root resistance to *Phytophthora palmivora*

## Graphical Abstract

**Figure:**
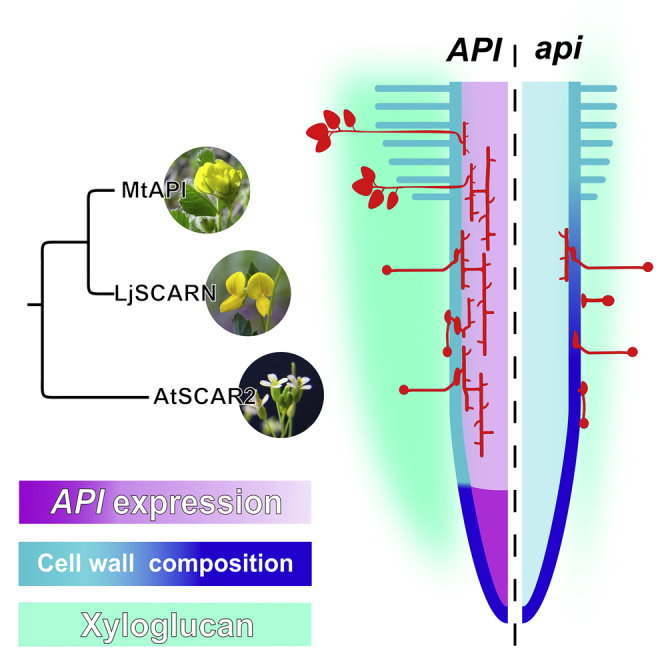

## Teaser

Subtly altered plant cell walls can be decisive for disease. Gavrin et al. show that the Medicago SCAR/WAVE complex protein API controls actin cytoskeleton dynamics in roots. Local changes associated with a loss of *API* establish a cell wall architecture with altered biochemical properties that hinder infection progress by an oomycete pathogen.

## Introduction

The cell wall protects plant cells from microbial invasion while maintaining properties enabling growth and development. Cell wall structures and modifications, such as the waxy cuticle and lignification, provide mechanical barriers for entry attempts [1]. Additionally, reinforcement of the cell wall through the deposition of carbohydrates results in the formation of papilla structures at attempted penetration sites [2]. Despite these measures, adapted pathogens have evolved strategies to ensure their passage through the cell wall. This raises the important question to what extent selective plant cell wall architecture changes can result in pathogen resistance while maintaining other biologically important properties, such as normal growth [3].

*Phytophthora palmivora* belongs to a genus of aggressive hemibiotrophic pathogens causing diseases in many important tropical crops [4]. *P. palmivora* has a wide host spectrum and is able to infect root and leaf tissues of several plant species, ranging from liverworts [5] to monocotyledonous flowering plants [6] and including legumes widely used in symbiosis research [7]. During root infection, mobile *P. palmivora* zoospores accumulate just above the root cap [8], where they encyst and form germ tubes with terminal appressoria to penetrate the subapical root epidermis and rapidly colonize the root cortex. Entry is facilitated in part through localized secretion of plant-cell-wall-degrading enzymes [9]. In the cortex, *P. palmivora* grows mostly intercellularly and projects short specialized hyphae, termed haustoria, through the walls of individual living root cells, resulting in the invagination of their protoplast. This is followed by a necrotrophic stage, characterized by host tissue necrosis and the formation of sporangia, which release new zoospores for further infection [10]. Unlike pathogenic interactions where cell wall modifications may block microbial entry, symbiotic interactions rely on cell wall remodeling to guide microbial entry and facilitate the establishment of nutrient exchange interfaces [11]. Rhizobia infection of roots of model legumes, such as *Medicago truncatula* and *Lotus japonicus*, occurs via host-driven tubular tip-growing structures known as infection threads (ITs). These tubular structures are initiated within curled root hairs following a two-step process, which involves localized host cell wall remodeling before IT polar tip growth initiates and progresses along the root hair length [12]. Mutational analyses in *M. truncatula* and *L. japonicus* have revealed that targeted secretion of cell wall polysaccharides, local degradation of plant cell walls, and cytoskeleton rearrangements are required for normal initiation and progression of ITs [13, 14, 15, 16, 17, 18, 19].

Plant cell wall biosynthesis relies on cellular secretory processes and the cytoskeleton. Major structural components of the primary walls are cellulose, hemicelluloses, and pectins. The polysaccharides, remodeling proteins, and some biosynthetic machinery that generate the cell walls are delivered through endomembrane trafficking [20, 21]. Cellulose is synthesized at the plasma membrane by a membrane-deployed cellulose synthase complex, whereas hemicelluloses and pectins are synthesized in the Golgi by sequential modification of the side chains in the various Golgi cisternae. The transport of synthesized polysaccharides to the plasma membrane is generally considered to be mediated by the default exocytosis pathway [22]. Xyloglucan is the most abundant hemicellulose in the primary cell wall of angiosperms, where it binds to cellulose microfibers and may link them together [23]. Furthermore, xyloglucan is secreted by root cells into the surrounding soil, where it acts as an efficient soil aggregator [24].

Deposition of pectins and hemicelluloses at the cell wall is largely dependent on the dynamics of the actin cytoskeleton, which at a whole-cell spatial scale dictates the patterns of intracellular transport and secretion [22]. Therefore, actin filaments directly affect plant cell wall establishment and remodeling. The formation and maintenance of the actin network is largely dependent on actin-binding proteins and actin filament nucleators. In plants, formins and ARP2/3 (actin-related protein 2/3) are two important actin filament nucleators, which confer actin filament branching. The ARP2/3 complex, in its active conformation, nucleates actin filaments from the sides of an existing filament and initiates a daughter filament at a 70° angle [25]. Plant ARP2/3 activation relies solely on the heteromeric five-subunit SCAR/WAVE (suppressor of cyclic AMP [cAMP] Receptor/Wiskott-Aldrich syndrome protein-family Verprolin homologous protein) regulatory complex [26]. The SCAR/WAVE complex contributes to diverse processes and aspects of plant development, including asymmetric cell divisions and cell morphology [27, 28], trichome architecture, plant cell adhesive properties [29], root rigidity, cell-cell junction [30], and rhizobial infection thread progression [15, 16, 18].

SCAR proteins constitute core elements of the SCAR/WAVE complex. Their N-terminal SCAR homology domain (SHD) appears to regulate protein stability and their assembly into the SCAR/WAVE complex [31], although their C-terminal WCA/VCA (WA) domain is sufficient for ARP2/3 activation [32]. *Arabidopsis* encodes four SCARs, each with a similar domain organization [33]. Mutations in *AtSCAR2* are sufficient to cause a mild distorted trichome phenotype [29, 34]. The overlapping expression patterns of the four *Arabidopsis SCAR* genes and the relatively weak phenotypes of *scar2* mutant lines suggest potential functional redundancy among respective family members [35]. Although a role for SCAR/WAVE proteins has been reported in the context of plant symbiosis [16] and in pathogenic interactions in animals [36], their importance for plant-pathogen interactions has not been addressed.

To identify genetic components commonly required for the accommodation of pathogenic and symbiotic microbes, we carried out root oomycete infection assays on *M. truncatula* mutant seedlings affected in rhizobia-root colonization. We demonstrate that the Medicago *API* gene, as well as its *L. japonicus* and *Arabidopsis* orthologs, can control cell wall properties required for efficient Medicago root entry by the oomycete pathogen *P. palmivora*. *API* encodes a SCAR2 protein, a subunit of the plant SCAR/WAVE actin regulatory complex. Quantitative resistance to *P. palmivora* is likely attributable to a modified plant cell wall architecture, rather than an altered defense response transcriptome. We demonstrated impaired actin and endomembrane trafficking dynamics in *api* mutants, resulting in the distortion of secreted cell wall remodeling factors. This leads to changes in biochemical properties of root cell walls that likely impair pathogen root entry without affecting overall plant growth. Our work demonstrates that alterations in the cell wall architecture of specific root cells contribute to disease resistance during a compatible interaction without compromising root growth, offering a potential route to quantitative root resistance against *Phytophthora.*

## Results

### Plants Mutated in the SCAR2 Gene *API* Are Compromised in Root Entry of a Pathogenic Oomycete

**Figure 1:**
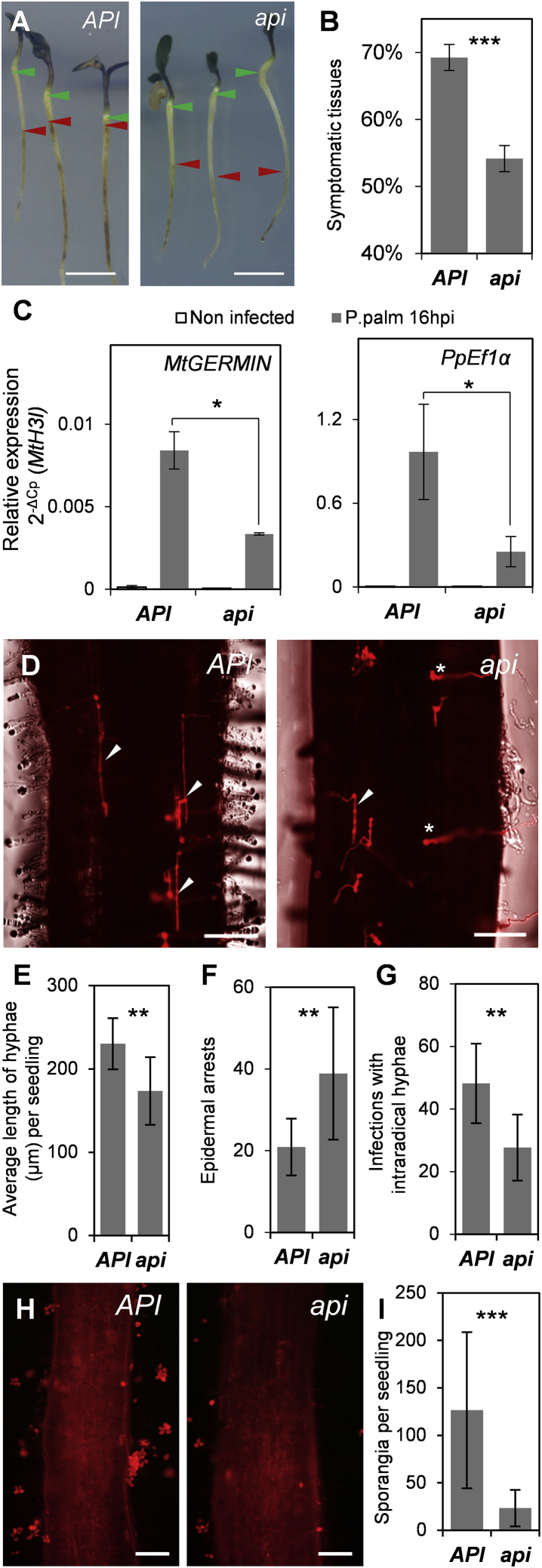
The *M. truncatula api* Mutant Is Resistant to Root Cellular Entry of the Pathogenic Oomycete *P. palmivora* (A) Visual disease symptoms of *API* (wild-type) and *api* seedlings after root tip infection with *P. palmivora* AJ-td zoospores at 72 h post-inoculation (hpi). Red arrowheads mark the extent of infection symptoms toward hypocotyl (green arrowhead); scale bars, 1 cm. (B) Scoring of visual disease symptom extent (symptom length/seedling length) of *API* (n = 58) and *api* (n = 48) plants inoculated with *P. palmivora* AJ-td at 72 hpi (error bars represent SEM; t test: ^∗∗∗^p < 0.001). (C) Quantification of the plant immunity marker *MtGERMIN* and *P. palmivora* biomass marker *PpEf1α* at 16 hpi by qRT-PCR using the 2^−ΔCp^ method and *MtH3l* as a reference gene (error bars represent SEM; n = 3, t test: ^∗^p < 0.05). (D) Epifluorescence microscopy of *API* and *api* seedlings at 12 hpi with *P. palmivora* AJ-td. Arrowheads indicate infections with intraradical growing hyphae. Asterisks indicate infections limited to the epidermis cell; scale bars, 100 μm. See also Figure S1A. (E) Average length of all *P. palmivora* AJ-td intraradical hyphae per seedling at 12 hpi (seedlings analyzed: n = 10 per genotype; error bars represent SD; t test: ^∗∗^p < 0.01). (F) Total amount of infections at the stage of the epidermis cell penetration per seedling at 12 hpi (seedlings analyzed: n = 10 per genotype; error bars represent SD; t test: ^∗∗^p < 0.01). (G) Total amount of infections with intraradical hyphae per seedling at 12 hpi (seedlings analyzed: n = 10 per genotype; error bars represent SD; t test: ^∗∗^p < 0.01). (H) Epifluorescence microscopy of *API* and *api* seedlings at 4 dpi with *P. palmivora* LILI-td. Scale bars, 150 μm. (I) Total amount of sporangia per seedling at 4 dpi (seedlings analyzed: n = 25 per genotype; error bars represent SD; t test: ^∗∗∗^p < 0.001). See also Figures S1 and S2.

When surveying mutants impaired in symbiosis [10] for their ability to resist pathogen infection, we found that seedling roots carrying the *api* (altered nodule primordia invasion) mutation [37] displayed significantly reduced disease symptoms upon infection with *P. palmivora* zoospores. Visual symptoms, pathogen biomass, and defense gene activation were reduced in the *api* mutant (Figures 1A–1C). Microscopic inspection revealed that *P. palmivora* infections were hindered at the root entry stage and infectious intraradical hyphae were shorter and less frequent in *api* compared to wild type (Figures 1D–1G). Reduced penetration was not isolate specific and was consistently observed with both the *P. palmivora* AJ-td strain from Indonesia as well as with LILI-td originating from Colombia, which also formed haustoria in *api* mutants (Figure S1A). At 4 days post inoculation (dpi), *P. palmivora* LILI-td produced significantly less sporangia on *api* mutants compared to wild type (Figures 1H and 1I). Taken together, these results indicate that the *api* mutant is compromised in *P. palmivora* colonization and sporulation.

**Figure 2:**
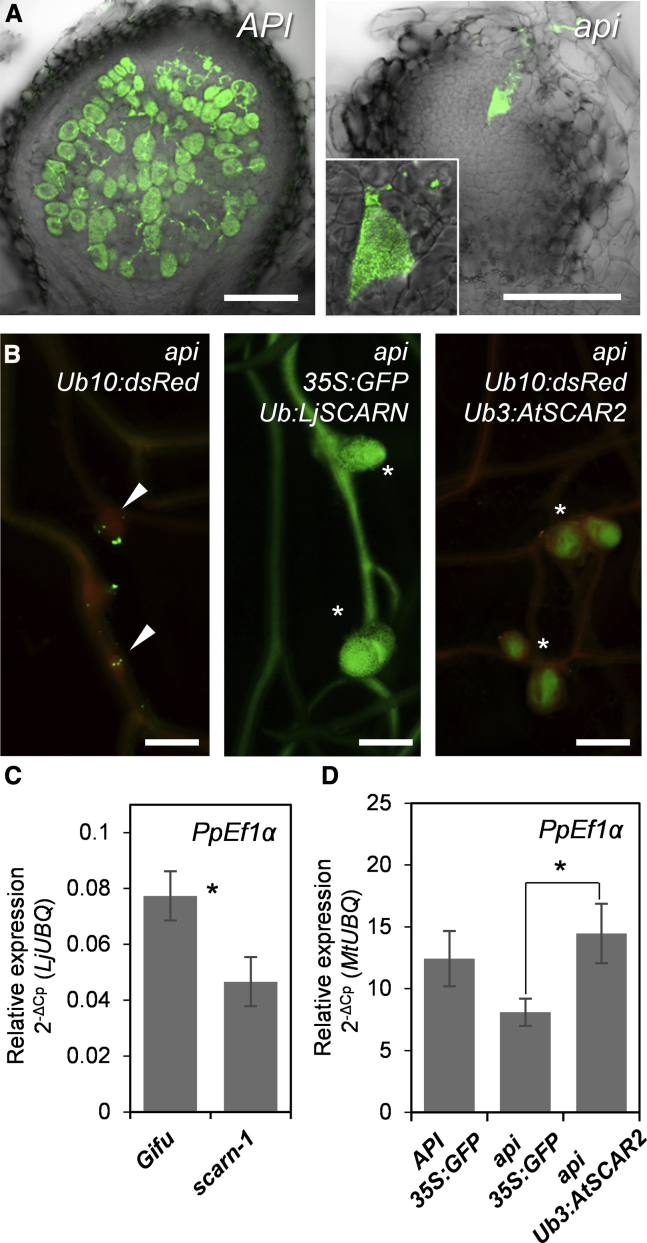
SCAR2-Related Proteins of Different Plant Species Can Support Rhizobia and *P. palmivora* Infection (A) Microscopy of *API* and *api* nodules at 18 days post-inoculation (dpi) with GFP-expressing *Sinorhizobium meliloti* 2011 (plants analyzed: n = 20 per genotype; scale bars, 150 μm). (B) Epifluorescence microscopy of root nodules in *api* hairy roots expressing *Ub10:dsRed*, *Ub:LjSCARN*, and *Ub3:AtSCAR2*. Asterisks indicate fully developed bacteria-filled nodules at 18 dpi; arrowheads indicate non-invaded outgrowths with arrested infection foci on top (green fluorescence); scale bars, 1 mm. See also Figure S3D. (C) Quantification of the *P. palmivora* AJ-td biomass marker *PpEf1*α in wild-type *L. japonicus* Gifu and *scarn-1* mutant by qRT-PCR using the 2^−ΔCp^ method and *LjUBQ* as a reference gene (error bars represent SD; biological replicates n = 3; t test: ^∗^p < 0.05). (D) Quantification of the *P. palmivora* LILI-YKDel biomass marker *PpEf1*α in *API* and *api* transgenic roots expressing *35S:GFP* (controls) and *api* roots expressing *Ub3:AtSCAR2* by qRT-PCR using the 2^−ΔCp^ method and *MtUBQ* as a reference gene (error bars represent SD; biological replicates n = 5; t test: ^∗^p < 0.05). See also Figures S2 and S3E.

The *api* mutant was initially reported as rhizobia-infection defective, resulting in the frequent development of non-invaded underdeveloped nodule primordia with large infection pockets and a reduced root hair length phenotype [37]. We confirmed the rhizobia-infection defective phenotype of *api* using fluorescently labeled *Sinorhizobium meliloti* bacteria. Overall, in our conditions, we found that 70% of *api* nodules are non-invaded outgrowths where bacteria accumulated in micro-colonies between cells of the root nodule primordia (Figure 2A).

To identify the genetic basis of the observed cell entry phenotypes, we combined Illumina sequencing of DNA from wild-type and mutant genotypes with transcriptome expression analyses (Data S1A). The previously genetically mapped target interval [37] was further narrowed down (Figure S1B). We identified a G2324A mutation in the SCAR2 protein-encoding gene MtrunA17_Chr4g0004861 (Medtr4g013235) that results in an early stop codon (Figure S1C). Transcript levels of MtrunA17_Chr4g0004861were attenuated in the mutant in both Affymetrix microarray data and quantitative reverse transcriptase PCR (qRT-PCR) analysis (Data S1A; Figure S2D). The gene encoded by MtrunA17_Chr4g0004861 was termed *API* in accordance with the previously described genetic locus [37]. In SCAR/WAVE complex mutants, leaf trichomes and overall development are frequently altered [15, 29, 38]. By contrast, we did not observe any changes in overall development, seed production, or trichomes between wild-type and *api* mutants besides the reduced root hair length (Figures S1E–S1H), and previously reported leaf chlorosis and reduced root growth were attributed to a reduced nitrogen fixing ability [37]. Microscopic investigation of sectioned and toluidine-blue-stained roots suggests that *api* mutants overall display a normal cellular architecture. In only 4 of 30 investigated roots, we observed small groups of cells with altered organization, which, however, did not impact on overall root development (Figures S1I–S1P).

Expression of the *API* gene in transgenic roots of *api* under *Ubiquitin 3* (*Ub3*) promoter or a 3.5-kb upstream sequence restored root hair length, wild-type frequencies of bacterial-colonized nodules, and full susceptibility to *P. palmivora* root infection as measured by pathogen biomass, respectively (Figures S2A–S2I). To obtain independent evidence for the role of *API* in *P. palmivora* infection, we used a hairpin RNAi construct targeting the 3′ UTR of MtrunA17_Chr4g0004861 (*hpAPI*) to attenuate *API* transcript levels in wild-type roots. Roots expressing the *hpAPI* construct displayed the shorter root hair phenotype (Figures S2J and S2K), in contrast to control roots expressing a construct targeting the non-existent *uidA* gene (*hpuidA*). We further confirmed that *hpAPI-*expressing transgenic roots infected with *P. palmivora* showed a significant reduction in *API* transcripts concomitantly with a reduction of *P. palmivora* biomass markers (Figure S2L). Taken together, the data demonstrate that the SCAR2 protein API is required for efficient colonization of *M. truncatula* by the filamentous pathogen *P. palmivora* and symbiotic rhizobia and *api* plants do not display altered overall plant development.

### SCAR2 Functionality Is Maintained in Different Plant Species

API belongs to a small family of SCAR-related proteins found across plant species (Figure S3A). The *L. japonicus API* ortholog *LjSCARN* has been proposed to function in nodulation [16]. However, when expressed under ubiquitin- or epidermis-specific *EXPA7* promoter [39] in roots of composite plants, *SCARN* can complement all *M. truncatula api* mutant root phenotypes (Figures 2B, S3B, and S3C), suggesting that SCARN’s functionality is not restricted to root nodulation but is also involved in an oomycete pathogen interaction. In support, *L. japonicus scarn-1* mutants were also more resistant to *P. palmivora* root infection (Figure 2C). Furthermore, ubiquitin-promoter-driven expression of *AtSCAR2*, the closest homolog of *API* from *Arabidopsis*, was also able to complement all *api* mutant phenotypes (Figures 2B, 2D, S3D, and S3E). Therefore, SCAR2-related proteins of different plant species can support rhizobia and *P. palmivora* infection in *M. truncatula* as well as in *L. japonicus*.

### *API* and Its Homologs Are More Strongly Expressed in Root Meristems and Nodule Primordia Tissues

**Figure 3:**
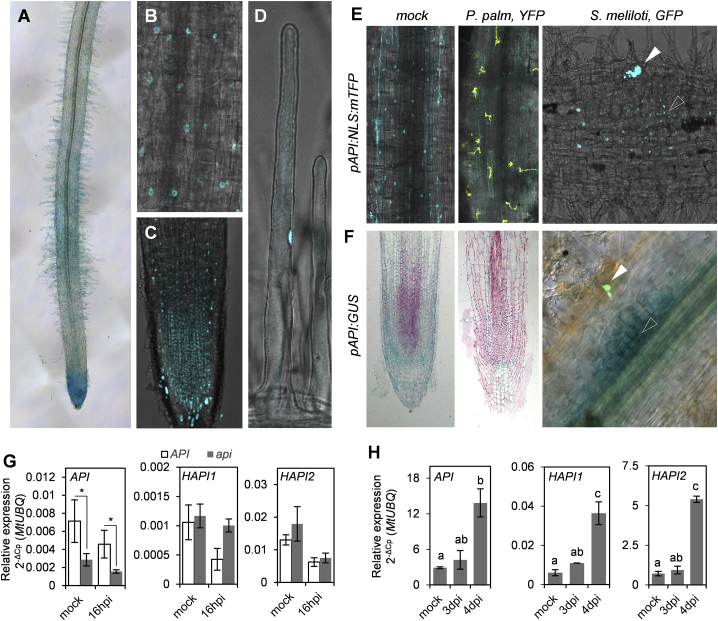
*SCAR* Genes Are Preferentially Expressed in Root Meristems and Nodule Primordia (A) GUS staining of uninfected transgenic Medicago roots expressing a *pAPI:GUS* reporter. (B–D) Expression of a *pAPI:NLS:mTFP* reporter in epidermal cells of the maturation zone (B), meristem and elongation zone (C), and a root hair cell (D). (E) Medicago roots expressing a *pAPI:NLS:mTFP* reporter at mock, 24 h upon *P. palmivora* LILI-YKDel infection, and 4 days post-inoculation with GFP expressing *S. meliloti*. Open arrowhead indicates dividing cells of a nodule primordium; closed arrowhead indicates an infection thread. (F) GUS staining of transgenic Medicago roots expressing *pAPI:GUS* reporter at mock, 24 h upon *P. palmivora* LILI-YKDel infection, and 4 days post-inoculation with GFP expressing *S. meliloti*. Open arrowhead indicates dividing cells of a nodule primordia; closed arrowhead indicates an infection thread. (G) Transcript levels of *API*, *HAPI1*, and *HAPI2* genes upon *P. palmivora* AJ-td inoculation in *API* and *api* background. qRT-PCR was analyzed using 2^−ΔCp^ method and *MtUBQ* as a reference gene (error bars represent SD; biological replicates n = 3; t test: ^∗^p < 0.05). (H) Transcript levels of *API*, *HAPI1*, and *HAPI2* genes at early stages of root nodule development in wild-type background. qRT-PCR was analyzed using 2^−ΔCp^ method and *MtUBQ* as a reference gene (error bars represent SD; biological replicates n = 3; one-way ANOVA with post hoc Tukey HSD test p < 0.05). See also Figures S4 and S5 and Data S1 and S2.

We investigated the expression patterns of *API* and its close homologs *HAPI1* (homolog of *API* 1) and *HAPI2* in transgenic roots of Medicago composite plants by examining the activity of transcriptional 2-kb-promoter fusions to GUS (β-glucuronidase) or a nuclear localized mTFP (monomeric teal fluorescent protein) and by employing qRT-PCR. GUS activity was mainly detectable in the meristematic root tip region, in root hairs, and in dividing cells of nodule primordia (Figures 3A and 3F). The mTFP reporter confirmed this expression in the root tips and nodule primordia (Figures 3C and 3E) as well as in root epidermal cells and root hairs (Figures 3B and 3D). At that stage, we could not detect significantly elevated levels of GUS activity associated with infection thread progression or at sites of *P. palmivora* infection. Instead, GUS and mTFP signals appeared weaker in pathogen-infected roots (Figures 3E and 3F). However, qRT-PCR did not show a statistically significant reduction. *API* transcript levels remained unaltered during *P. palmivora* infection (Figure 3G) but increased over time during nodule primordia development (Figure 3H). *HAPI1* and *HAPI2* showed similar expression patterns in infected and non-infected roots as well as in young developing nodule primordia (Figures 3G, 3H, and S4). In the *api* mutant background, we did not detect compensating higher transcript levels of *HAPI1* and *HAPI2* (Figure 3G). Together, these data suggest that Medicago *SCAR* genes are not induced upon early *P. palmivora* infection and their upregulation during nodule development is likely attributable to cell division activity of nodule primordia rather than linked to infection thread progression in the cortex.

### Induced Responses to Pathogen Infection Are Unaltered in *api* Mutants despite Impaired Actin Dynamics

We next addressed whether *api* mutants display an altered transcriptional response to *P. palmivora* infection. Microarray experiments indicated only minor changes in transcript levels between uninfected wild-type and mutant seedlings: just 44 of the 50,900 probes hybridized differentially to 32 assignable transcripts (log2FC > 1; Data S1A; Figure S5A). This indicates that the *api* mutation does not lead to massive constitutive deregulation of the transcriptome. Just a single probe matching a putative transcript of the Mapman biotic stress category [40] was differentially expressed with a log2FC = 1.05, illustrating an absence of a constitutively heightened defense response. Infection by *P. palmivora* AJ-td deregulated a more significant set of transcripts (Data S1B). However, the infection resulted in similar overall transcriptome dynamics when comparing wild-type and *api* seedlings (Figure S5B) with just 65 differential probes between both genotypes, four of them assigned to “biotic stress” genes (Data S1C).

Overall similar microarray gene expression profiles were further confirmed by qRT-PCR analysis, and similar levels of previously published pathogen-induced transcripts *PRX*, *GLP*, *LOX*, and *THA* [41, 42] were quantified in both mutant and wild-type contexts (Figure S5C; Data S2).

Because SCAR2 proteins have been reported to regulate actin processes [35], we assessed whether actin responses were affected in the *api* mutant background upon microbial infection. Confocal fluorescence microscopy of wild-type and *api* mutant roots expressing the ABD2-YFP (actin binding domain2-yellow fluorescent protein) actin reporter did not reveal changes in density and bundling of actin in cortical cells of root areas targeted by *P. palmivora* (Figures S5D–S5F). *P. palmivora* penetration sites of *api* and wild-type seedlings showed similar levels of closely positioned plant nuclei (Figure S5G), indicating no significant impairment of actin-mediated nuclear repositioning in *api*. Furthermore, no difference in actin bundle distribution was observed in developing root nodules of both wild-type and mutant plants (Figure S5H).

**Figure 4:**
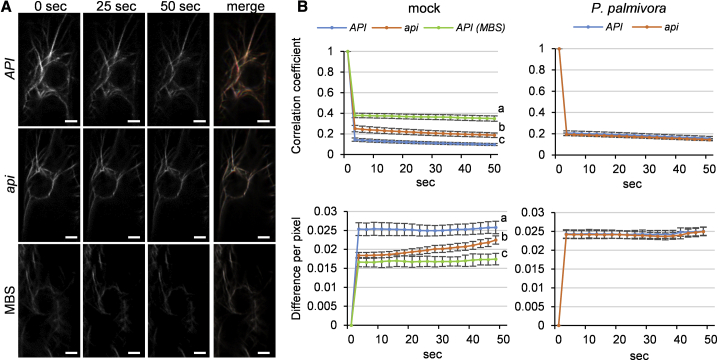
The Elongation Zone of *api* Roots Displays Impaired Actin Dynamics (A) Representative images from a single focal plane time-lapse series at 2.5-s intervals of *API* and *api* hairy root epidermal cells expressing YFP-ABD2 actin reporter. The merged image (far-right column) shows all three time points as separate color channels in a red, green, and blue (RGB) image. MBS-treated cells exhibit very minor changes in actin filament organization over the time course analyzed, and this is represented by an almost completely white overlay in the merged image. Scale bars represent 3 μm. (B) Quantification of actin dynamics in *API* and *api* background upon infection with *P. palmivora* and mock conditions using two independent methods: total differences per pixel and correlation coefficient (ten transgenic roots were analyzed for each condition; cells analyzed: mock n_*API*_ = 27, n_*api*_ = 26; infected n_*API*_ = 23, n_*api*_ = 41; error bars represent SE; two-way ANOVA with post hoc Tukey HSD test p < 0.05). See also Figure S5.

However, *api* mutants are quantitatively impaired in actin dynamics in epidermal and cortical cells of the root elongation zone (Figure 4). This is revealed by consecutive time-lapse images showing a higher correlation coefficient and a lower pixel difference in *api* mutants than in *API* wild type (Figures 4A and 4B). Importantly, *api* mutants still retained the ability for dynamic actin changes compared to roots treated with the inhibitor MBS (m-maleimidobenzoyl-N-hydroxysuccinimide ester), and actin dynamics upon *P. palmivora* infection are not different between *api* and wild type (Figure 4B). Taken together, although transcriptomic changes associated to defense responses were similar in wild-type and *api* roots, *api* mutants display impaired actin dynamics but remain unaltered in actin network density and bundling.

### API Controls Endomembrane Trafficking to Establish Cell Wall Properties Compatible with Infection Processes

**Figure 5:**
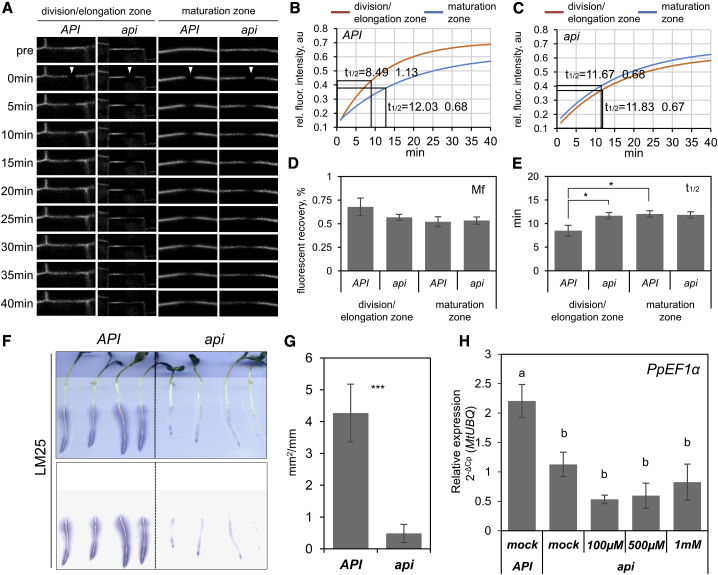
*api* Mutants Display Delayed PIP1 Plasma Membrane Protein Recovery and Reduced Secretion of Xyloglucan (A) Fluorescence recovery after photobleaching within 40 min measured in inner root cells of elongation and maturation zones of *API* and *api* transgenic roots expressing Ub3:PIP1-GFP. (B and C) Fluorescence recovery kinetics of PIP1-GFP in *API* (B) and *api* (C) transgenic roots (*API*: n_*root tip*_ = 11, n_*mature root*_ = 11; *R*^2^_*root tip*_ = 0.95, *R*^2^_*mature root*_ = 0.97; *api:* n_*root tip*_ = 12, n_*mature root*_ = 10; *R*^2^_*root tip*_ = 0.95, *R*^2^_*mature root*_ = 0.99). (D) Quantification of the mobile fraction contributing to fluorescence recovery of PIP1-GFP at the membrane measured in different root areas of *API* and *api* transgenic roots expressing *Ub3:PIP1-GFP* (error bars represent SE; number of FRAP: root tip n_*API*_ = 11, n_*api*_ = 12; mature root n_*API*_ = 11, n_*api*_ = 10). (E) Quantification of the halftime of recovery in different root areas of *API* and *api* transgenic roots expressing *Ub3:PIP1-GFP* (error bars represent SE; number of FRAP root tip n_*API*_ = 11, n_*api*_ = 12; mature root n_*API*_ = 11, n_*api*_ = 10; t test: ^∗^p < 0.05). (F) Immunodetection of xyloglucan (LM25) secretion from plant surfaces. Bright-field image of *API* and *api* seedlings grown on agar solid media superimposed with the nitrocellulose print of the solid media surface after removal of seedlings is shown, which was then probed with LM25 antibodies (n = 10 per genotype). See also Figure S6C. (G) Quantification of LM25 antibody signal (error bars represent SD; biological replicates n = 4 per genotype; t test: ^∗∗∗^p < 0.001). (H) Quantification of the *P. palmivora* biomass in xyloglucan-treated infected seedlings by qRT-PCR using the 2^−ΔCp^ method and *MtUBQ* as a reference gene (error bars represent SE; biological replicates n = 3; one-way ANOVA with post hoc Tukey HSD test p < 0.05).

To investigate whether altered actin dynamics in *api* result in quantitative differences in secretory processes, we monitored how fast the plasma membrane integral aquaporin PIP1-GFP fusion is redeployed after photobleaching (Figure 5A). The recovery of PIP1-GFP fluorescence after photobleaching was significantly reduced in cells of division and elongation zones of *api* roots, but no difference was observed in generally slower recovering mature root cells (Figures 5B–5E). This suggests that altered endomembrane dynamics are restricted to those root tissues that display pronounced activity. We subsequently monitored brefeldin-A (BFA)-induced BFA-body formation in the outer cortex of *api* root elongation zones over time using a GFP secretion reporter system [43]. These analyses revealed that accumulation of GFP-labeled endomembrane compartments into a BFA body was delayed in *api*, resulting in the formation of small BFA bodies (Figures S6A and S6B). This delay was not caused by differences in general chemical permeability between wild-type and *api* mutants (Figure S6A).

We then tested whether the delayed endomembrane dynamics in *api* may affect the secretion of specific cell wall components. An immunoblot of root secretions probed with monoclonal antibodies (mAbs) toward specific cell wall components indicated a reduction in secreted xyloglucans in *api* mutants relative to wild-type *API* controls, as detected by the xyloglucan-specific antibody LM25 (Figures 5F and 5G). In contrast, no differences were observed when using antibodies toward pectic polysaccharides of homogalacturonan (via LM19) or RG-I (via LM5, LM26, and LM6-M; Figure S6C). These results suggest a role for SCAR2 proteins in cell wall xyloglucan secretion. Nevertheless, the external application of xyloglucans to *api* seedlings did not restore susceptibility to *P. palmivora* (Figure 5H). Taken together, *api* roots display delayed endomembrane compartment trafficking and altered selective secretion of root cell wall components.

**Figure 6:**
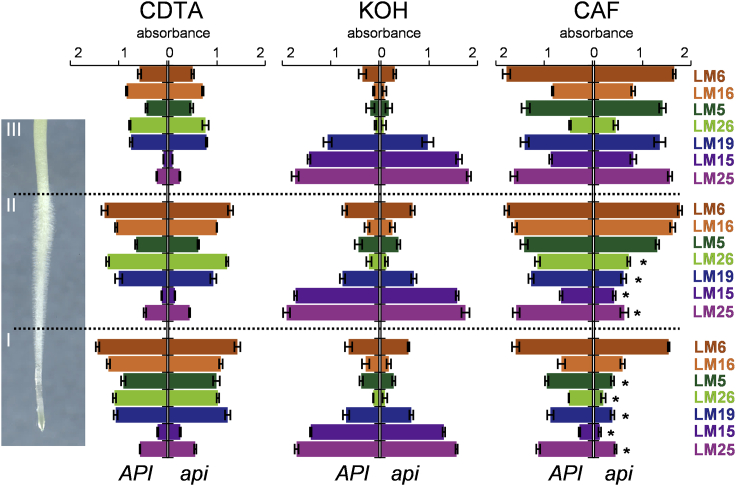
Cell Walls of *api* Display Altered Biochemical Properties ELISA analysis of cell wall polysaccharides extracted sequentially with CDTA, KOH, and cellulase treatment yielding the cellulose-associated fraction (CAF) from three root developmental zones: comprising (I) the root meristem and elongation zone; (II) the root hair differentiation zone; and (III) the mature root zone of *API* and *api* roots (error bars represent SD; biological replicates n = 3; t test: ^∗^p < 0.05). See also Figure S7.

We next addressed whether *api* root cell walls exhibited altered polysaccharide composition. Wall extracts from three developmental zones of seedling roots (regions I to III in Figure 6) were analyzed via enzyme-linked immunosorbent assay (ELISA) using a range of mAbs toward cell wall polysaccharides (see STAR Methods). No alteration in composition or abundance of any of the tested cell wall components was detected within fractions extracted with calcium chelating (CDTA) and alkaline buffers (KOH), and in support, immunolocalization of different epitopes in cross sections did not reveal any conspicuous differences (Figures 6 and S7). However, solubilization of the cellulose microfibrils and their tightly associated polysaccharides as a cellulose-associated fraction (CAF) [44] revealed an *api*-specific reduction of epitopes recognized by mAbs toward xyloglucans (LM15 and LM25), RG-I (LM5 and LM26), and homogalacturonan (LM19) in the specific zones I and II comprising meristematic, elongation, and root hair differentiation zones of the root (Figure 6). This transient decrease in detectable cellulose-associated pectin and xyloglucan in the *api* mutants suggests an alteration in the cell wall architecture in root zones where the preferential colonization by *P. palmivora* takes place.

**Figure 7:**
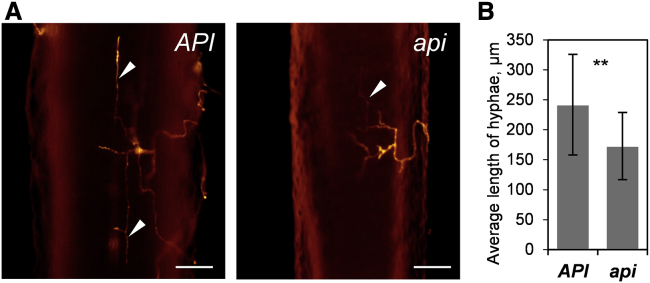
*P. palmivora* Infection of Chloroform-Treated *API* and *api* Plants (A) Epifluorescence microscopy of chloroform-treated *API* and *api* seedlings 20 hpi after inoculation with *P. palmivora* LILI-td. Arrowheads indicate intraradical growing hyphae; scale bars, 100 μm. (B) Average length of *P. palmivora* LILI-td intraradical hyphae per seedling (error bars represent SD; seedlings analyzed: n = 14 per genotype; t test: ^∗∗^p < 0.01).

Our data collectively revealed significant differences in actin dynamics, endomembrane secretion processes, cell wall biochemistry, and architecture in *api* mutants relative to wild-type Medicago plants. To specifically assess the contribution of altered cell wall properties rather than actin dynamics, endomembrane trafficking, and secretion processes to the pathogen phenotype, we infected seedlings that were chemically inactivated using chloroform treatment. At 20 h post-application of spores, *P. palmivora* hyphae colonizing these dead seedlings were shorter in *api* mutants compared to wild type (Figure 7). Thus, *P. palmivora* colonization of *api* root tissues is impaired even if these tissues are dead. This suggests that *API* mediates establishment of a preformed cell wall architecture that supports full seedling colonization by this oomycete. By contrast, *api* mutants present a subtly altered cell wall architecture that is quantitatively more resistant.

## Discussion

We have identified a premature stop codon mutation in the SCAR2-protein-encoding gene *API* as being responsible for increased quantitative resistance to *P. palmivora* root infection through the modulation of cell wall properties relevant for infection. A *L. japonicus SCARN* gene mutation has previously been found to alter root hair length and to impair rhizobia infection during root nodule symbiosis in *L. japonicus*. On the basis of phylogenetic analyses, the authors had suggested that SCARN may have arisen from a gene duplication and acquired specialized functions in root nodule symbiosis [16]. We demonstrate here that SCAR2-mediated phenotypes in *M. truncatula* and *L. japonicus* are not limited to nitrogen fixing symbiosis but also impact root susceptibility to an oomycete pathogen. Therefore, *API* constitutes a gene commonly contributing to symbiotic and pathogenic microbe colonization in different plants. Furthermore, we showed that ectopic expression of *Arabidopsis SCAR2* was able to complement all observed *api* mutant phenotypes, suggesting that legumes have not evolved additional functionality for SCAR2 proteins during rhizobial colonization.

Notably, mutation of *RIT* (required for infection thread), encoding the core SCAR/WAVE complex component NAP1 (Nck-Associated Protein 1), results in an arrest of rhizobia infection threads at earlier stages of colonization and visible changes in aboveground trichome development in *M. truncatula* [15]. By contrast, *api* mutants did not display such dramatic developmental defects (Figures S1E and S1F). Although infection threads are partially defective in *api*, they can still initiate and progress through root hairs [37], even though nodule primordia colonization is strongly impaired. It is tempting to speculate that different members of the SCAR family integrate into different SCAR/WAVE complexes consisting of the other components, including NAP1, and therefore, loss of function of a single *SCAR* gene only impairs a subset of cell processes. *API* is stronger expressed in root meristems, and PIP1-GFP recovery after photobleaching was faster in these tissues. It is plausible that *API* may contribute more in tissues with a higher demand in cytoskeleton-mediated trafficking processes.

*SCAR/WAVE* mutants often display very specific and restricted phenotypes, such as trichome changes [29] or root hair length (Figure S2F), and higher order *Arabidopsis* SCAR/WAVE gene mutants are not massively impaired in all development processes [35]. Cell morphological changes in *api* are limited to shorter root hairs and rare events of altered cell groups (Figure S1). It is likely that overall root development is thus maintained through the activity of remaining *API* homologs as well as other actin regulators. Future work will aim at elucidating whether and how different SCAR proteins confer specific functions in plant-microbe interactions as well as growth and development in specific tissues. An alternative, albeit less likely, possibility for the absence of any dramatic developmental defects in *api* could be its residual transcript levels (Figure S1D). Nonetheless, this would result in the expression of a truncated protein lacking the essential C-terminal domains that confer the link to the ARP2/3 complex.

Previous studies have highlighted the potential role of cell-wall components to support pathogen infection [3], but the underlying mechanisms remain to be clarified. We demonstrated that the cellulose-associated fractions of *api* root meristematic, elongation, and differentiation zones display reduced levels of detectable epitopes of xyloglucan (LM15 and LM25), RG-I (LM5 and LM26), and homogalacturonan (LM19). A reduction of these polysaccharides in the cellulose-associated fraction of *api* suggests an altered cell wall architecture in these particular root zones. Reduced tethering of xyloglucan and pectic carbohydrates during synthesis of cellulose microfibrils is expected to alter the wall’s rheological behavior [45] and influence pathogen penetration. The defective incorporation of non-cellulosic polysaccharides into cellulose microfibrils in *api* is likely caused by changes in cytoskeleton-mediated endomembrane compartment dynamics, as visualized through impaired actin dynamics and delayed membrane protein delivery, as well as delayed BFA body formation (Figures 4, 5, and S6). This likely impacts on the secretory compartment system for delivering cell wall pectins, xyloglucans, and associated remodeling enzymes. Therefore, our data support a link between API SCAR/WAVE protein-mediated actin cytoskeleton rearrangements, secretory processes, and the extracellular matrix/cell wall composition, ultimately affecting microbial entry at root elongation and differentiation zones.

*SCAR* genes are expressed constitutively during infection, but the activity of promoter reporter gene fusions was higher in actively dividing tissues of the root tip and nodule primordia (Figure 3). These actively dividing tissues have a high demand for cell wall material deployment. It is therefore reasonable that a mutation in *api* has a stronger impact in these tissues. Delayed endomembrane dynamics in *api* roots lead to an extended area with altered cell wall properties compared to wild type (Figure 6), reaching into the root hair emergence zone, where rhizobial symbiotic colonization takes place. Importantly, *P. palmivora* zoospores preferentially accumulate at root elongation zones [43, 46], where *api* mutants display altered cell wall properties. It is tempting to speculate that microbial infections in mature root sections remain unaltered, contributing to the quantitative nature of the phenotype. Consistently, no significantly altered arbuscular mycorrhizal fungal symbiosis was observed in *api* mutants [37]. A benefit of such developmental stage-specific resistance is that plants are resilient to pathogens targeting this specific region, such as *P. palmivora*, without being impaired in their overall root system, shoot size, and seed production. Reported plant resistance strategies against *P. palmivora* include the expression of pathogenicity-related genes and antimicrobial secondary metabolites [47]. Furthermore, a diversity panel survey in *M. truncatula* failed to identify completely resistant accessions. Instead, polymorphisms near *RAD1* were correlated with the extent of root disease symptoms [8]. These works and our study highlight the importance of quantitative disease resistance in the control of this pathogen.

## STAR★Methods

### Key Resources Table

**Table:** 

REAGENT or RESOURCE	SOURCE	IDENTIFIER
**Antibodies**		

LM5 Rat monoclonal	[48]	N/A
LM6 Rat monoclonal	[49]	N/A
LM11 Rat monoclonal	[50]	N/A
LM15 Rat monoclonal	[51]	N/A
LM16 Rat monoclonal	[52]	N/A
LM19 Rat monoclonal	[52]	N/A
LM20 Rat monoclonal	[52]	N/A
LM23 Rat monoclonal	[53]	N/A
LM24 Rat monoclonal	[53]	N/A
LM25 Rat monoclonal	[53]	N/A
LM26 Rat monoclonal	[44]	N/A
Rat IgG (whole molecule)-Peroxidase antibody produced in rabbit	Sigma-Aldrich	Cat# A9542; RRID:AB_258456
Rat IgG (whole molecule)-FITC antibody produced in rabbit	Sigma-Aldrich	Cat# F1763; RRID:AB_259443

**Bacterial and Virus Strains**		

*Agrobacterium rhizogenes* arqua 1	Lab stocks	N/A
*Agrobacterium rhizogenes* AR1193	Lab stocks	N/A
*E. coli* TOP10 Chemically Competent	C404010	N/A
*Sinorhizobium meliloti* 2011	[54]	N/A

**Chemicals, Peptides, and Recombinant Proteins**		

Brefeldin A	Sigma-Aldrich	Cat# B6542
m-maleimidobenzoyl-N-hydroxylsuccinimide ester	Thermo Scientific	Cat# 22311

**Deposited Data**		

Microarray data	http://www.ncbi.nlm.nih.gov/geo/query/acc.cgi?acc=GSE65903	GSE65903
Sequencing data	http://www.ddbj.nig.ac.jp	DRA003037
Sequencing data	https://www.ncbi.nlm.nih.gov/sra	SRR8468999
Sequencing data	Bioproject	PRJNA516327

**Experimental Models: Organisms/Strains**		

*Medicago truncatula* Jemalong A17	Lab stocks	N/A
*M. truncatula* Jemalong A17 *api* EMS mutant line	[37]	N/A
*Lotus japonicus* Gifu B-129	[16]	N/A
*L.* japonicus *scarn-1* EMS mutant line	[16]	N/A
*Phytophthora palmivora* AJ-td	Lab stocks	N/A
*Phytophthora palmivora* LILI-td	Lab stocks	N/A
*Phytophthora palmivora* LILI-YKDel	Lab stocks	N/A

**Oligonucleotides**		

Primers for plasmid construction see Table S1	Eurofins	N/A

**Recombinant DNA**		

pUB-GW-GFP-pUb:SCARN	[16]	N/A
pUB-GW-GFP-pEp:SCARN	[16]	N/A
pUC57-API	GENEWIZ	N/A
pENTR:pAPI	This paper	N/A
pENTR:pHAPI1	This paper	N/A
pENTR:pHAPI2	This paper	N/A
pENTR:AtSCAR2	This paper	N/A
pKGW-MGW-pAPI:API	This paper	N/A
pKGW-MGW-pUb:API	This paper	N/A
pKGW-MGW-pUb:AtSCAR2	This paper	N/A
pK7GWIWG2-35S:hpAPI	This paper	N/A
pKGW-GGRR-pHAPI1:GUS	This paper	N/A
pKGW-GGRR-pHAPI2:GUS	This paper	N/A
pKGW-MGW-pAPI:NLS:mTFP	This paper	N/A
pKGW-MGW-pAPI:GUS	This paper	N/A
pCAMBIA1300-35S:YFP-ABD2	This paper	N/A
pK7WGF2-pUb:SPpr1-GFP	[43]	N/A
pKGW-MGW-pUb:PIP1-GFP	This paper	N/A

**Software and Algorithms**		

MATLAB function corr2	[55]	N/A
Actin_difference_maps	This paper	N/A
Actin_skewness-and-occupancy	This paper	N/A
Microsoft Excel	Microsoft Office	N/A
AxioVision SE64 Rel. 4.9.1	N/A	N/A
RStudio Version 1.0.44	https://www.rsudio.com	N/A
R 3.0.2	https://www.r-project.org/	N/A
Mega6	https://www.megasoftware.net/	N/A
AGCC Scan Control Software	http://www.affymetrix.com/	N/A
GeneSpring GX 12.6.1	https://www.agilent.com/	N/A
MAPMAN BINs	https://mapman.gabipd.org/	N/A
BWA software	http://bio-bwa.sourceforge.net/	N/A
Coval software	https://omictools.com/coval-tool	N/A
MATLAB	https://www.mathworks.com/	N/A
Fiji	[56]	N/A

### Lead Contact and Materials Availability

Further information and requests for resources and reagents should be directed to and will be fulfilled by the Lead Contact, Sebastian Schornack (sebastian.schornack@slcu.cam.ac.uk). All unique strains and plasmids used for this paper are available from the Lead Contact with a completed Materials Transfer Agreement.

### Experimental Model and Subject Details

#### Plant materials and microbial strains

*Medicago truncatula* Jemalong A17 wild-type (referred as *API*) seeds were kindly provided by Dr. Jean-Marie Prospéri (INRA-Montpellier). The *api* mutant used in this study is derived from the *M. truncatula* Jemalong A17 reference line after EMS mutagenesis and two backcrosses and displayed similar germination rate and seedling morphology as wild-type [37]. Medicago seed sterilization, germination and growth conditions have been described previously [10]. *Agrobacterium rhizogenes-*based root transformation of Medicago was performed according to Limpens et al. [57]. For the nodulation assay Medicago roots were inoculated with GFP-expressing *Sinorhizobium meliloti* 2011 [54].

To allow tracking of the oomycete development *in planta* fluorescently labeled *Phytophthora palmivora* AJ-td (derived from accession P6390) and LILI-td (derived from accession P16830) carrying a pTOR:TdTomato vector or LILI-YKDel carrying a pTOR:CALYFP-KDEL were used for the infection assays. *P. palmivora* isolates were cultivated on V8 vegetable juice agar plates and used to infect *M. truncatula* plants and to phenotype them as described previously [10]. Briefly, zoospores were released in cold water. The spore concentration was adjusted to 5x10^4^ spores ml^–1^. 10 μl droplets of *P. palmivora* AJ-td or LILI-td spores were placed at the root tip of M. *truncatula* seedlings on 0.8% agarose plates. In each infection experiment a single application of zoospores was carried out. The same protocol was used for chloroform-treated seedling infection and xyloglucan supplemented infection. For chloroform treatment 1-day-old seedlings were soaked for 10 min in chloroform, rinsed several times with sterile water and infected on 0.8% agarose plates. High purity xyloglucan (Megazyme) was used to prepare a 50mM stock solution in deionized warm water. To obtain a working concentration of xyloglucan the necessary amount of stock solution was added directly to the spore suspension. Spore motility was checked microscopically before infection.

### Method Details

#### Genetic mapping and SNPs analysis

Previous work indicated that the *api* root hair and symbiotic phenotype are caused by a single recessive mutation, and the *API* locus was mapped to a 2.8 cM interval on the upper arm of *M. truncatula* linkage group 4 between markers MTIC331 and McSSR1 [37]. For finer mapping of the locus we screened 193 individuals of the F2 progeny from a cross between the *M. truncatula api* mutant (backcrossed twice in the A17 genetic background) and the *M. truncatula* wild-type line F83005.5. We identified 8 lines with recombination events in the above-defined interval between markers MTIC331 and EPJ005 and tested their symbiotic phenotype in F2 and F3 generations. Using 6 additional molecular markers located between MTIC331 and EPJ005 based on *M. truncatula* genome sequence, we were able to position the *API* locus to an interval of about 550 kb (Figure S2A). To identify the *api* mutation we sequenced the *api* mutant (accession SRR8468999, Bioproject: PRJNA516327) and in parallel employed a comparative genome sequencing strategy. *API* and *api* plant genomic DNA was extracted using DNeasy Plant Mini Kit (QIAGEN). The libraries for whole genome sequencing were prepared with TruSeq DNA LT Sample Prep Kit (Illumina) and subjected to 100 bp paired-end sequencing by Illumina Hiseq2500. Raw data can be found on http://www.ddbj.nig.ac.jp/ using accession *DRA003037*. The sequence reads in which more than 10% of sequenced nucleotides had a phred quality score of less than 30 were excluded from the subsequent analysis. For detecting SNPs of *api*, we first constructed a “reference sequence” of *API* by replacing the nucleotides of the publicly available A17 *M*. *truncatula* reference genome (ftp://ftp.jcvi.org/pub/data/m_truncatula/Mt4.0/Assembly/JCVI.Medtr.v4.20130313.fasta) with those of *API* as described previously [58]. A total of 122,523 SNP positions were substituted. The sequence reads from *api* were aligned to the developed *API* reference sequence using BWA software [59]. Alignment files were subjected to filtering using Coval software [60] and calculating the SNP-index for all SNP positions using the MutMap pipeline [61]. Finally the SNP positions having a sequence depth > 5 and showing an SNP-index > 0.9 were extracted as homozygous SNPs of *api*. Presence of the *api* causal mutation was confirmed via Sanger sequencing service at Source Bioscience (http://www.sourcebioscience.com/).

#### Design of constructs and cloning

Coding sequences of *API* (Medtr4g013235/MtrunA17_Chr4g0004861) were synthesized in pUC57 vector by GENEWIZ, Inc. Coding sequences of *AtSCAR2* (AT2G38440) and *PIP1* (Medtr8g098375/MtrunA17_Chr8g0386961) were amplified from *Arabidopsis thaliana* (Columbia-0 ecotype) and *M. truncatula* A17 cDNA, respectively. 2kb of 5′ regulatory sequences of *API*, *HAPI1* (Medtr7g071440/MtrunA17_Chr7g0244031) and *HAPI2* (Medtr8g086300/MtrunA17_Chr8g0379031) were amplified from *M. truncatula* A17 genomic DNA. PCR reactions were performed using Phusion DNA polymerase (New England Biolab Inc., UK) and primers listed in Table S1. Amplicons were introduced into pENTR (D-TOPO® Cloning Kit, Thermo Fisher Scientific) and used as an entry vectors. To generate complementation constructs entry vectors containing clones of *API* and *AtSCAR2* were recombined with pENTR:prAtUBQ3 into pKGW-MGW destination vector [62] using LR Clonase Plus (Thermo Fisher Scientific). To generate the *PIP1-GFP* reporter construct, the *PIP1* entry vector was recombined into a pK7FWG2 destination vector. pENTR clones of promoters were recombined into a pKGW-GGRR destination vector, creating promoter GUS or mTFP fusions using LR Clonase Plus (Thermo Fisher Scientific).

#### Microscopy

For imaging of *P. palmivora* colonization in infected *API* and *api* root sections excised root tissues were mounted in water and covered by coverslips, using a Leica TCS SP8 confocal microscope with emission/excitation settings 561/570-600 nm for the *P. palmivora* AJ-td and 514/520-550 nm for the *P. palmivora* LILI-YKDel strain. Images represent stacks of 20 to 25 1-μm slices in maximum intensity projections merged with inverted transillumination images to outline cells. A line averaging of 4 was applied to reduce noise to signal ratio. Epifluorescence microscopy and sporangia counting were carried out using a Fluorescent Stereo Microscope Leica M165 FC equipped with a DFC310FX camera. The DSR filter (10447412) was used to detect the tdTomato produced by *P. palmivora* LILI-td or AJ-td. The same settings were used to screen for transgenic hairy roots of *M. truncatula* expressing monomeric DsRED. Light microscopy imaging and infection scoring were performed using Zeiss Axioimager M2 microscope with a 64 MP color camera. Pictures were processed with ImageJ software v1.46 including Fiji plugins and AxioVision Microscopy Software (Zeiss).

#### Promoter GUS assay

Medicago transgenic roots were collected and washed twice in 0.1 M sodium phosphate buffer, pH 7.2, incubated in GUS buffer (100 mM sodium phosphate pH 7.0, 5 mM K_3_Fe(CN)_6_, 5 mM K_4_Fe(CN)_6_ and 2 mM 5-bromo-4-chloro-3-indoxyl-β-d- glucuronic acid, X-gluc) under vacuum at room temperature for 30 min to allow the buffer to replace air in the tissue, incubated at 37°C for 2-6 h to enable the enzymatic reaction, and analyzed using the Microscope Leica M165 FC and Zeiss Axioimager M2 microscope.

#### Quantification of actin dynamics and organization

Medicago hairy roots expressing a YFP-ABD2 actin reporter were used for analysis of actin filaments. Imaging was conducted using a Leica SP8 confocal microscope. Images were acquired at 1024 × 1024-pixel resolution. Individual cells were imaged every 2.5 s over a 60 s time course. MBS (m-maleimidobenzoyl N-hydroxylsuccinimide ester) was used as actin stabilizing treatment [63]. Twenty to forty cells were used to calculate actin filament dynamics following the protocols in Vidali et al. [55]. Briefly, the algorithm calculates the correlation and difference of each time-lapse frame by averaging correlation and difference between this frame and all other frames. The correlation was defined as two-dimensional correlations between two matrixes, and the difference was considered by subtracting two matrixes. Correlation coefficient was calculated using the built-in MATLAB function corr2 [55] and differences per pixel were calculated using Actin_difference_maps (https://github.com/SchornacklabSLCU/Actin_difference_maps). For quantification of actin filament bundling and occupancy, cells were imaged using 1024 × 1024 pixel resolution. A maximum intensity projection of 15–20 image stacks was subjected for image processing and analysis performed as described by Higaki et al. [64]. The serial optical sections of the root cell images were skeletonized using the ‘Analyze Skeleton (2D/3D)’ plugin in Fiji [56, 65] and projected. Actin bundling was estimated as the skewness of the intensity distribution of the actin filament pixels. Filament occupancy was defined as ratio of the total pixel numbers of actin filaments and cell area using Actin_skewness-and-occupancy (https://github.com/SchornacklabSLCU/Actin_skewness-and-occupancy).

#### FRAP acquisition and analysis

Medicago hairy roots expressing a PIP1-GFP reporter were used for fluorescence recovery after photobleaching (FRAP) analysis. Root samples were mounted in water on microscope slides and sealed with Valap (vaseline:lanolin:paraffin, melted and combined 1:1:1). Root cells were imaged using the Leica FRAP wizard. Three scans were made to establish the pre-bleach intensity and then a circular region of interest (2 μm diameter) was drawn in an optical section of the plasma membrane. Twenty iterations of 488 nm set at 100% laser power were used for bleaching. Recovery of the fluorescence was recorded during 40 min with a delay of 1 min between frames and a resolution of 512 × 512 pixels. FRAP analysis was performed according to Rademacher et al. [66].

#### Endomembrane dynamics analysis

Medicago hairy roots expressing SPPR1-GFP reporter [43] were treated with 50 μM brefeldin A (BFA). The GFP fluorescence signal intensity and distribution were analyzed after 1-2h of treatment. For image processing and analysis, the software ImageJ was used. To obtain intensity information for each BFA compartment per cell, a picture of the GFP channel was duplicated and thresholded to highlight all structures of interest. Once the areas are highlighted a binary version of the image was created. Subsequent steps involved setting the “Redirect to” line to the name of the image of GFP channel and selecting the checkboxes “Mean grey value” and “Area.” The “Analyze Particles” tool then obtained mean pixel intensities and area for the BFA compartments on the original image, based on outlines applied from the binary image.

#### Cell wall extraction, ELISA and immunolabelling

Three developmental root zones were harvested in triplicate from 2-day old seedlings of wild-type and *api* Medicago plants, frozen in liquid nitrogen, ground in a tissue lyser, and converted into an alcohol insoluble residue (AIR) [67] via successive washes with 80%, 90% and 100% (v/v) ethanol, acetone and finally methanol:chloroform (2:3 v/v) and left to dry overnight.

The AIRs were extracted sequentially with 50 mM CDTA (1,2-cyclohexanediamine tetraacetic acid, pH 7.5) and 4 M KOH. Residue remaining after the KOH extract was further extracted to solubilise polysaccharides tightly associated with cellulose via chemical solubilisation [44] to yield the cellulose associated fractions (CAF).

CDTA, KOH and CAF fractions were diluted in PBS (1 in 20 for CDTA and KOH, 1 in 5 for CAF), coated onto microtiter plates (Nunc, maxisorb), and ELISAs were performed as described [67]. MAbs used in this study are toward homogalacturonan LM19 and LM20 [52]; RG-I LM5 [48]; LM6-M [49]; LM16 [52]; LM26 [44], xyloglucan LM15 [51]; LM24 and LM25 [53]; xylan LM11 [50], and xylosyl residues LM23 [53].

Root samples used for microscopy were fixed overnight in phosphate-buffered saline (PBS) containing 4% paraformaldehyde and 1% glutaraldehyde. Samples were then washed twice in PBS before being dehydrated in an ethanol series of 5, 10, 15, 20, 30, 40, 50, 60, 70, 80, 90, and twice 100% at 4°C. Samples were then embedded in LR White resin, and polymerized at 37°C. Resin-embedded material was then sectioned (1 μm thickness). Sections were incubated in PBS containing 5% (w/v) milk protein (MP/PBS) and a 5-fold dilution of antibody hybridoma supernatant for 1.5 h. Samples were then washed in PBS at least 3 times and incubated with a 100-fold dilution of anti-rat-IgG-whole molecule-FITC (Sigma-Aldrich, F1763) in MP/PBS for 1.5 h in darkness. The samples were washed in PBS at least 3 times and incubated with Calcofluor White 0.2 μg/mL (Sigma-Aldrich, 18909) for 5 min in darkness. Samples were washed at least 3 times and then mounted in a glycerol-based anti-fade solution (Citifluor AF1, Agar Scientific, UK).

#### Gene expression assays

RNA extraction was performed with 100 mg of pooled plant material for each biological replicate using RNeasy Mini Kit (QIAGEN), reverse transcription of first strand cDNA and qPCR analysis were performed and analyzed as described in Rey et al. [10]. Medicago *MtH3l* and *MtUBQ* genes were used as reference genes to standardized expression of *M. truncatula* genes of interest in seedlings and hairy roots [41]. In addition, qRT-PCR was also used to assess the accumulation of *P. palmivora* biomass by tracking expression of the oomycete housekeeping genes of *Elongation factor 1α* (*Ef1α*) identified by homology to *Phytophthora parasitica* transcripts [68]. The 2^-ΔCp^ method was used to display gene expression levels [69]. Primer sequences used in this study are listed in Table S1.

#### Microarray

Affymetrix Medicago Gene Chip hybridizations were carried out in biological triplicates at IMGM (Germany). *M. truncatula* total RNAs were extracted as described previously and quality assessed using NanoDrop ND-1000 spectral photometer and a 2100 Bioanalyzer (Agilent Technologies). Total RNA was processed according to manufacturer recommendations using the 3′IVT Expression Kit (Affymetrix Inc.). After hybridization, microarrays signal intensities were detected with the Affymetrix GeneChip® 3000 Scanner and AGCC Scan Control Software v3.2.3.1515 (Affymetrix). Automatic grid was arranged and raw data (^∗^.DAT files) were processed to generate image and intensity files (^∗^:CEL, ^∗^.JPG) by the AGCC Scan Control Software. The software tool GeneSpring GX 12.6.1 (Agilent Technologies) was used for visualization and normalization (using the RMA algorithm). After normalization, data is returned as log2 transformed values. Significant differential expression level between *API* and *api* plants was assessed for each individual probe using a Student’s t test and a Bonferroni correction in R (3.0.2, ww.r-project.org). The Affymetrix probeset was annotated according to Czaja et al. [70] while MAPMAN BINs were used to classify probes in biological processes (http://mapman.gabipd.org/web/guest) and the probes were mapped to version V4.0 of the Medicago gene models (http://jcvi.org/medicago/) Raw data (.CEL files) can be found on the Gene Expression Omnibus (http://www.ncbi.nlm.nih.gov/geo/query/acc.cgi?acc=GSE65903).

### Quantification and Statistical Analysis

The experiments were performed in three independent replicates. The number of biological replicates, exact n values and the type of statistical significance test are described in the figure legends. Statistical significance was determined by ANOVA with Tukey’s post hoc test or the two-tailed Student’s t test.

### Data and Code Availability

Microarray data can be found on the Gene Expression Omnibus: GSE65903. Sequencing data can be found on the Bioinformation and DDBJ Center: DRA003037, The Sequence Read Archive: SRR8468999, and Bioproject: PRJNA516327. The Actin_difference_maps script is available on https://github.com/SchornacklabSLCU/Actin_difference_maps. The Actin_skewness-and-occupancy script is available on https://github.com/SchornacklabSLCU/Actin_skewness-and-occupancy.

## Supplemental Information

Document S1. Figures S1–S7

Table S1. Primers Used in This Study, Related to STAR Methods

Data S1. List of Probes Showing Differential Regulation between *API* and *api* Plants and at 16 h after Infection, Related to Figures 3 and S5 (A) List of probes showing differential regulation between wild type *API* plants and *api* mutant plants in control condition. Significantly differential probes with a log2 ratio > 1 are in red, repressed probes are in green (log2 ratio < −1), data were considered significant when the fold change lead to a Bonferroni adjusted p value < 0.05. Affymetrix GeneChip Medicago Transcriptome Assay probes identifiers are in column A. Gene accession numbers from version 4.0 of the *M. truncatula* genome are in column B. Mapman Mapping Mt_AFFY_Mt3.0_0510 of each gene is indicated in column C and D. Annotations are displayed in column G. (B) List of probes showing differential regulation between *API* plants at 16 hours post inoculation compared to *API* mock plants as well as *api* plants at 16 hours post inoculation compared to *api* mock plants. Significantly differential probes with a log2 ratio > 1 are in red, repressed probes are in green (log2 ratio < −1), data were considered significant when the fold change lead to a Bonferroni adjusted p value < 0.05. Affymetrix GeneChip Medicago Transcriptome Assay probes identifiers are in column A. Gene accession numbers from version 4.0 of the *M. truncatula* genome are in column B. Mapman Mapping Mt_AFFY_Mt3.0_0510 of each gene is indicated in column C and D. Annotations are displayed in column I. (C) List of probes showing differential regulation between *API* and *api* plants at 16 hours post inoculation. Significantly differential probes with a log2 ratio > 1 are in red, repressed probes are in green (log2 ratio < −1), data were considered significant when the fold change lead to a Bonferroni adjusted p value < 0.05. Affymetrix GeneChip Medicago Transcriptome Assay probes identifiers are in column A. Gene accession numbers from version 4.0 of the *M. truncatula* genome are in column B. Mapman Mapping Mt_AFFY_Mt3.0_0510 of each gene is indicated in column C and D. Annotations are displayed in column G.

Data S2. Validation of Microarray Expression Values by RT-qPCR, Related to Figures 3 and S5 Expression level was measured by RT-qPCR using the 2^-ΔCp^ method using *MtH3l* as reference gene. Subsequently, gene regulation between mock and 16 hpi with *P. palmivora* (*API* 16hpi / *API* mock and *api* 16 hpi / *api* mock) and between genotypes (*api* mock / *API* mock and *api* 16hpi / *API* 16hpi) was determined using the 2^-ΔΔCp^ method. Gene expression ratios obtained from microarray (array) and qPCR were plotted on a logarithmic scale (base 2). Similar gene induction at 16 hpi as compared to mock plants was observed in *API* and *api* both in RT-qPCR assays and array data for the immune marker genes *THA*, *PRX*, *GLP* and *LOX*. Genes showing significant fold change in a given ratio either in the array (Bonferroni-corrected p value < 0.05) or RT-qPCR (t test < 0.05) are colored in red when their log2 FC > 1 or in green when their log2 FC < −1.

Document S2. Article plus Supplemental Information

## References

[1] Yeats T.H., Rose J.K.C. (2013). The formation and function of plant cuticles. Plant Physiol..

[2] Bellincampi D., Cervone F., Lionetti V. (2014). Plant cell wall dynamics and wall-related susceptibility in plant-pathogen interactions. Front. Plant Sci..

[3] van Schie C.C., Takken F.L.W. (2014). Susceptibility genes 101: how to be a good host. Annu. Rev. Phytopathol..

[4] McHau G.R.A., Coffey M.D. (1994). Isozyme diversity in Phytophthora palmivora: evidence for a southeast Asian centre of origin. Mycol. Res..

[5] Carella P., Gogleva A., Tomaselli M., Alfs C., Schornack S. (2018). *Phytophthora palmivora* establishes tissue-specific intracellular infection structures in the earliest divergent land plant lineage. Proc. Natl. Acad. Sci. USA.

[6] Le Fevre R., O’Boyle B., Moscou M.J., Schornack S. (2016). Colonization of barley by the broad-host hemibiotrophic pathogen *Phytophthora palmivora* uncovers a leaf development-dependent involvement of *Mlo*. Mol. Plant Microbe Interact..

[7] Wang E., Schornack S., Marsh J.F., Gobbato E., Schwessinger B., Eastmond P., Schultze M., Kamoun S., Oldroyd G.E.D. (2012). A common signaling process that promotes mycorrhizal and oomycete colonization of plants. Curr. Biol..

[8] Rey T., Bonhomme M., Chatterjee A., Gavrin A., Toulotte J., Yang W., André O., Jacquet C., Schornack S. (2017). The *Medicago truncatula* GRAS protein RAD1 supports arbuscular mycorrhiza symbiosis and *Phytophthora palmivora* susceptibility. J. Exp. Bot..

[9] Meijer H.J.G., Mancuso F.M., Espadas G., Seidl M.F., Chiva C., Govers F., Sabidó E. (2014). Profiling the secretome and extracellular proteome of the potato late blight pathogen *Phytophthora infestans*. Mol. Cell. Proteomics.

[10] Rey T., Chatterjee A., Buttay M., Toulotte J., Schornack S. (2015). Medicago truncatula symbiosis mutants affected in the interaction with a biotrophic root pathogen. New Phytol..

[11] Rich M.K., Schorderet M., Reinhardt D. (2014). The role of the cell wall compartment in mutualistic symbioses of plants. Front. Plant Sci..

[12] Fournier J., Teillet A., Chabaud M., Ivanov S., Genre A., Limpens E., de Carvalho-Niebel F., Barker D.G. (2015). Remodeling of the infection chamber before infection thread formation reveals a two-step mechanism for rhizobial entry into the host legume root hair. Plant Physiol..

[13] Gavrin A., Chiasson D., Ovchinnikova E., Kaiser B.N., Bisseling T., Fedorova E.E. (2016). VAMP721a and VAMP721d are important for pectin dynamics and release of bacteria in soybean nodules. New Phytol..

[14] Hossain M.S., Liao J., James E.K., Sato S., Tabata S., Jurkiewicz A., Madsen L.H., Stougaard J., Ross L., Szczyglowski K. (2012). *Lotus japonicus ARPC1* is required for rhizobial infection. Plant Physiol..

[15] Miyahara A., Richens J., Starker C., Morieri G., Smith L., Long S., Downie J.A., Oldroyd G.E.D. (2010). Conservation in function of a SCAR/WAVE component during infection thread and root hair growth in *Medicago truncatula*. Mol. Plant Microbe Interact..

[16] Qiu L., Lin J.S., Xu J., Sato S., Parniske M., Wang T.L., Downie J.A., Xie F. (2015). SCARN a novel class of SCAR protein that is required for root-hair infection during legume nodulation. PLoS Genet..

[17] Xie F., Murray J.D., Kim J., Heckmann A.B., Edwards A., Oldroyd G.E.D., Downie J.A. (2012). Legume pectate lyase required for root infection by rhizobia. Proc. Natl. Acad. Sci. USA.

[18] Yokota K., Fukai E., Madsen L.H., Jurkiewicz A., Rueda P., Radutoiu S., Held M., Hossain M.S., Szczyglowski K., Morieri G. (2009). Rearrangement of actin cytoskeleton mediates invasion of *Lotus japonicus* roots by *Mesorhizobium loti*. Plant Cell.

[19] Timmers A.C. (2008). The role of the plant cytoskeleton in the interaction between legumes and rhizobia. J. Microsc..

[20] Kim S.-J., Brandizzi F. (2016). The plant secretory pathway for the trafficking of cell wall polysaccharides and glycoproteins. Glycobiology.

[21] Schneider R., Hanak T., Persson S., Voigt C.A. (2016). Cellulose and callose synthesis and organization in focus, what’s new?. Curr. Opin. Plant Biol..

[22] Kim S.-J., Brandizzi F. (2014). The plant secretory pathway: an essential factory for building the plant cell wall. Plant Cell Physiol..

[23] Zheng Y., Wang X., Chen Y., Wagner E., Cosgrove D.J. (2018). Xyloglucan in the primary cell wall: assessment by FESEM, selective enzyme digestions and nanogold affinity tags. Plant J..

[24] Galloway A.F., Pedersen M.J., Merry B., Marcus S.E., Blacker J., Benning L.G., Field K.J., Knox J.P. (2018). Xyloglucan is released by plants and promotes soil particle aggregation. New Phytol..

[25] Blanchoin L., Amann K.J., Higgs H.N., Marchand J.-B., Kaiser D.A., Pollard T.D. (2000). Direct observation of dendritic actin filament networks nucleated by Arp2/3 complex and WASP/Scar proteins. Nature.

[26] Yanagisawa M., Zhang C., Szymanski D.B. (2013). ARP2/3-dependent growth in the plant kingdom: SCARs for life. Front. Plant Sci..

[27] Facette M.R., Park Y., Sutimantanapi D., Luo A., Cartwright H.N., Yang B., Bennett E.J., Sylvester A.W., Smith L.G. (2015). The SCAR/WAVE complex polarizes PAN receptors and promotes division asymmetry in maize. Nat. Plants.

[28] Zhou W., Wang Y., Wu Z., Luo L., Liu P., Yan L., Hou S. (2016). Homologs of SCAR/WAVE complex components are required for epidermal cell morphogenesis in rice. J. Exp. Bot..

[29] Basu D., Le J., El-Essal Sel.-D., Huang S., Zhang C., Mallery E.L., Koliantz G., Staiger C.J., Szymanski D.B. (2005). DISTORTED3/SCAR2 is a putative arabidopsis WAVE complex subunit that activates the Arp2/3 complex and is required for epidermal morphogenesis. Plant Cell.

[30] Dyachok J., Shao M.-R., Vaughn K., Bowling A., Facette M., Djakovic S., Clark L., Smith L. (2008). Plasma membrane-associated SCAR complex subunits promote cortical F-actin accumulation and normal growth characteristics in Arabidopsis roots. Mol. Plant.

[31] Kurisu S., Takenawa T. (2009). The WASP and WAVE family proteins. Genome Biol..

[32] Kollmar M., Lbik D., Enge S. (2012). Evolution of the eukaryotic ARP2/3 activators of the WASP family: WASP, WAVE, WASH, and WHAMM, and the proposed new family members WAWH and WAML. BMC Res. Notes.

[33] Frank M., Egile C., Dyachok J., Djakovic S., Nolasco M., Li R., Smith L.G. (2004). Activation of Arp2/3 complex-dependent actin polymerization by plant proteins distantly related to Scar/WAVE. Proc. Natl. Acad. Sci. USA.

[34] Zhang X., Dyachok J., Krishnakumar S., Smith L.G., Oppenheimer D.G. (2005). *IRREGULAR TRICHOME BRANCH*1 in Arabidopsis encodes a plant homolog of the actin-related protein2/3 complex activator Scar/WAVE that regulates actin and microtubule organization. Plant Cell.

[35] Zhang C., Mallery E.L., Schlueter J., Huang S., Fan Y., Brankle S., Staiger C.J., Szymanski D.B. (2008). Arabidopsis SCARs function interchangeably to meet actin-related protein 2/3 activation thresholds during morphogenesis. Plant Cell.

[36] Shi J., Scita G., Casanova J.E. (2005). WAVE2 signaling mediates invasion of polarized epithelial cells by Salmonella typhimurium. J. Biol. Chem..

[37] Teillet A., Garcia J., de Billy F., Gherardi M., Huguet T., Barker D.G., de Carvalho-Niebel F., Journet E.-P. (2008). *api*, A novel Medicago truncatula symbiotic mutant impaired in nodule primordium invasion. Mol. Plant Microbe Interact..

[38] Rao Y., Yang Y., Xu J., Li X., Leng Y., Dai L., Huang L., Shao G., Ren D., Hu J. (2015). EARLY SENESCENCE1 encodes a SCAR-LIKE PROTEIN2 that affects water loss in rice. Plant Physiol..

[39] Hayashi T., Shimoda Y., Sato S., Tabata S., Imaizumi-Anraku H., Hayashi M. (2014). Rhizobial infection does not require cortical expression of upstream common symbiosis genes responsible for the induction of Ca(2+) spiking. Plant J..

[40] Goffard N., Weiller G. (2006). Extending MapMan: application to legume genome arrays. Bioinformatics.

[41] Nars A., Rey T., Lafitte C., Vergnes S., Amatya S., Jacquet C., Dumas B., Thibaudeau C., Heux L., Bottin A., Fliegmann J. (2013). An experimental system to study responses of Medicago truncatula roots to chitin oligomers of high degree of polymerization and other microbial elicitors. Plant Cell Rep..

[42] Rey T., Nars A., Bonhomme M., Bottin A., Huguet S., Balzergue S., Jardinaud M.F., Bono J.J., Cullimore J., Dumas B. (2013). NFP, a LysM protein controlling Nod factor perception, also intervenes in Medicago truncatula resistance to pathogens. New Phytol..

[43] Evangelisti E., Gogleva A., Hainaux T., Doumane M., Tulin F., Quan C., Yunusov T., Floch K., Schornack S. (2017). Time-resolved dual transcriptomics reveal early induced Nicotiana benthamiana root genes and conserved infection-promoting Phytophthora palmivora effectors. BMC Biol..

[44] Torode T.A., O’Neill R., Marcus S.E., Cornuault V., Pose S., Lauder R.P., Kračun S.K., Rydahl M.G., Andersen M.C.F., Willats W.G.T. (2018). Branched pectic galactan in phloem-sieve-element cell walls: implications for cell mechanics. Plant Physiol..

[45] Gu J., Catchmark J.M. (2014). Roles of xyloglucan and pectin on the mechanical properties of bacterial cellulose composite films. Cellulose.

[46] Hickman C.J. (1970). Biology of Phytophthora zoospores. Phytopathology.

[47] Carella P., Gogleva A., Hoey D.J., Bridgen A.J., Stolze S.C., Nakagami H., Schornack S. (2019). Conserved biochemical defenses underpin host responses to oomycete infection in an early-divergent land plant lineage. Curr. Biol..

[48] Jones L., Seymour G.B., Knox J.P. (1997). Localization of pectic galactan in tomato cell walls using a monoclonal antibody specific to (1[->]4)-[beta]-D-galactan. Plant Physiol..

[49] Cornuault V., Posé S., Knox J.P. (2018). Disentangling pectic homogalacturonan and rhamnogalacturonan-I polysaccharides: evidence for sub-populations in fruit parenchyma systems. Food Chem..

[50] McCartney L., Marcus S.E., Knox J.P. (2005). Monoclonal antibodies to plant cell wall xylans and arabinoxylans. J. Histochem. Cytochem..

[51] Marcus S.E., Verhertbruggen Y., Hervé C., Ordaz-Ortiz J.J., Farkas V., Pedersen H.L., Willats W.G., Knox J.P. (2008). Pectic homogalacturonan masks abundant sets of xyloglucan epitopes in plant cell walls. BMC Plant Biol..

[52] Verhertbruggen Y., Marcus S.E., Haeger A., Ordaz-Ortiz J.J., Knox J.P. (2009). An extended set of monoclonal antibodies to pectic homogalacturonan. Carbohydr. Res..

[53] Pedersen H.L., Fangel J.U., McCleary B., Ruzanski C., Rydahl M.G., Ralet M.-C., Farkas V., von Schantz L., Marcus S.E., Andersen M.C.F. (2012). Versatile high resolution oligosaccharide microarrays for plant glycobiology and cell wall research. J. Biol. Chem..

[54] Limpens E., Franken C., Smit P., Willemse J., Bisseling T., Geurts R. (2003). LysM domain receptor kinases regulating rhizobial Nod factor-induced infection. Science.

[55] Vidali L., Burkart G.M., Augustine R.C., Kerdavid E., Tüzel E., Bezanilla M. (2010). Myosin XI is essential for tip growth in *Physcomitrella patens*. Plant Cell.

[56] Schindelin J., Arganda-Carreras I., Frise E., Kaynig V., Longair M., Pietzsch T., Preibisch S., Rueden C., Saalfeld S., Schmid B. (2012). Fiji: an open-source platform for biological-image analysis. Nat. Methods.

[57] Limpens E., Mirabella R., Fedorova E., Franken C., Franssen H., Bisseling T., Geurts R. (2005). Formation of organelle-like N2-fixing symbiosomes in legume root nodules is controlled by DMI2. Proc. Natl. Acad. Sci. USA.

[58] Takagi H., Abe A., Yoshida K., Kosugi S., Natsume S., Mitsuoka C., Uemura A., Utsushi H., Tamiru M., Takuno S. (2013). QTL-seq: rapid mapping of quantitative trait loci in rice by whole genome resequencing of DNA from two bulked populations. Plant J..

[59] Li H., Durbin R. (2009). Fast and accurate short read alignment with Burrows-Wheeler transform. Bioinformatics.

[60] Kosugi S., Natsume S., Yoshida K., MacLean D., Cano L., Kamoun S., Terauchi R. (2013). Coval: improving alignment quality and variant calling accuracy for next-generation sequencing data. PLoS ONE.

[61] Abe A., Kosugi S., Yoshida K., Natsume S., Takagi H., Kanzaki H., Matsumura H., Yoshida K., Mitsuoka C., Tamiru M. (2012). Genome sequencing reveals agronomically important loci in rice using MutMap. Nat. Biotechnol..

[62] Limpens E., Ivanov S., van Esse W., Voets G., Fedorova E., Bisseling T. (2009). Medicago N2-fixing symbiosomes acquire the endocytic identity marker Rab7 but delay the acquisition of vacuolar identity. Plant Cell.

[63] Sonobe S., Shibaoka H. (1989). Cortical fine actin filaments in higher plant cells visualized by rhodamine-phalloidin after pretreatment with m-maleimidobenzoyl N-hydroxysuccinimide ester. Protoplasma.

[64] Higaki T., Kutsuna N., Sano T., Kondo N., Hasezawa S. (2010). Quantification and cluster analysis of actin cytoskeletal structures in plant cells: role of actin bundling in stomatal movement during diurnal cycles in Arabidopsis guard cells. Plant J..

[65] Arganda-Carreras I., Fernández-González R., Muñoz-Barrutia A., Ortiz-De-Solorzano C. (2010). 3D reconstruction of histological sections: Application to mammary gland tissue. Microsc. Res. Tech..

[66] Rademacher D.J., Cabe M., Bakowska J.C. (2019). Fluorescence recovery after photobleaching of yellow fluorescent protein tagged p62 in aggresome-like induced structures. J. Vis. Exp..

[67] Torode T.A., Marcus S.E., Jam M., Tonon T., Blackburn R.S., Hervé C., Knox J.P. (2015). Monoclonal antibodies directed to fucoidan preparations from brown algae. PLoS ONE.

[68] Yan H.-Z., Liou R.-F. (2006). Selection of internal control genes for real-time quantitative RT-PCR assays in the oomycete plant pathogen Phytophthora parasitica. Fungal Genet. Biol..

[69] Livak K.J., Schmittgen T.D. (2001). Analysis of relative gene expression data using real-time quantitative PCR and the 2^(-Δ^^Δ C(T^)) method. Methods.

[70] Czaja L.F., Hogekamp C., Lamm P., Maillet F., Martinez E.A., Samain E., Dénarié J., Küster H., Hohnjec N. (2012). Transcriptional responses toward diffusible signals from symbiotic microbes reveal *MtNFP*- and *MtDMI3*-dependent reprogramming of host gene expression by arbuscular mycorrhizal fungal lipochitooligosaccharides. Plant Physiol..

