# Supplementary material for: Developmental Modulation of Root Cell Wall Architecture Confers Resistance to an Oomycete Pathogen
Source: Curr Biol. 2020 Nov 2;30(21):4165–4176.e5. doi: 10.1016/j.cub.2020.08.011 (PMC7658807; doi:10.1016/j.cub.2020.08.011)
Supplement: Document S1. Figures S1–S7 [file mmc1.pdf]

Current Biology, Volume *30*

Supplemental Information

Developmental Modulation of Root Cell Wall Architecture Confers Resistance to an Oomycete Pathogen

Aleksandr Gavrin, Thomas Rey, Thomas A. Torode, Justine Toulotte, Abhishek Chatterjee, Jonathan Louis Kaplan, Edouard Evangelisti, Hiroki Takagi, Varodom Charoensawan, David Rengel, Etienne-Pascal Journet, Frédéric Debellé, Fernanda de Carvalho-Niebel, Ryohei Terauchi, Siobhan Braybrook, and Sebastian Schornack

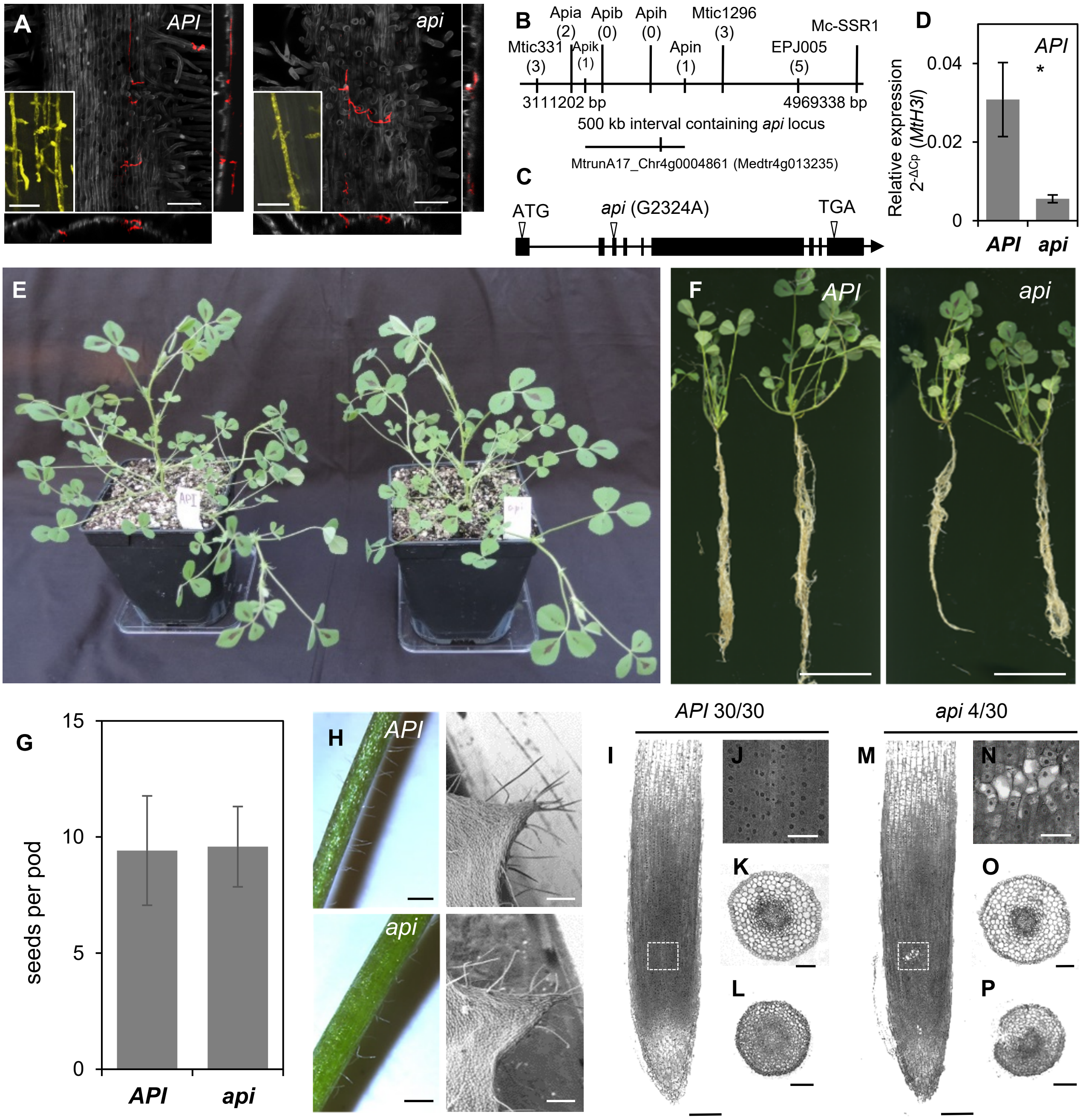

Figure S1. Mapping and phenotypes of *api* mutant plants. Related to Figure 1.

A, Microscopy of the root tip region of *API* and *api* seedlings at 12hpi with *P. palmivora* LILI-td (red). Intraradical hyphae can be observed in *API* while penetration is impaired in *api.* Insets display hyphae with haustoria 48 hours post infection with *P. palmivora* LILI-YKDEL (yellow). scale bars=50µm and 10 µm (insets). **B**, Physical/genetic map of the *API* locus. The molecular markers used for fine mapping of the *API* locus and the number of recombinants between the *API* locus and the marker are shown above the line. The position of the markers bordering the *API* region on the *M. truncatula* chromosome 4 (Mt 5.0 assembly) is shown below the line together with the interval containing the *API* locus. **C**, Position of a G to A substitution in MtrunA17_Chr4g0004861/Medtr4g013235 (*API*) in *api* plants resulting in an early stop codon in position 110 out of 1573 of the protein within the SCAR Homology Domain (SHD). **D**, Expression level of *API* gene in *API* and *api* plants using the $2^{-\Delta Cp}$ method and *MtH3l* as a reference gene (error bars represent SD; biological replicates n=3; t-test: *, p<0.05). **E, F** Overall *API* and *api* plant morphology after 4 weeks of growth in sand/soil mix, scale bars=5cm. **G,** Quantification of seeds production by *API* and *api* plants, **H,** Trichome morphology and density in *API* and *api* plants at the stem and leaf tips. Bars=1 mm for brightfield pictures and bars=200µm in scanning electronic microscopy pictures. **I,** A longitudinal section of *API* root. **J,** Magnification of **I**. **K,** A cross section of developed region of *API* root. **L,** A cross section of *API* root tip. **M,** A longitudinal section of *api* root. **N,** Magnification of **M**. **O,** A cross section of developed region of *api* root. **P,** A cross section of *api* root tip. For I and M scale bars=200µm. For J, K, L, N, O, P scale bars=100µm.

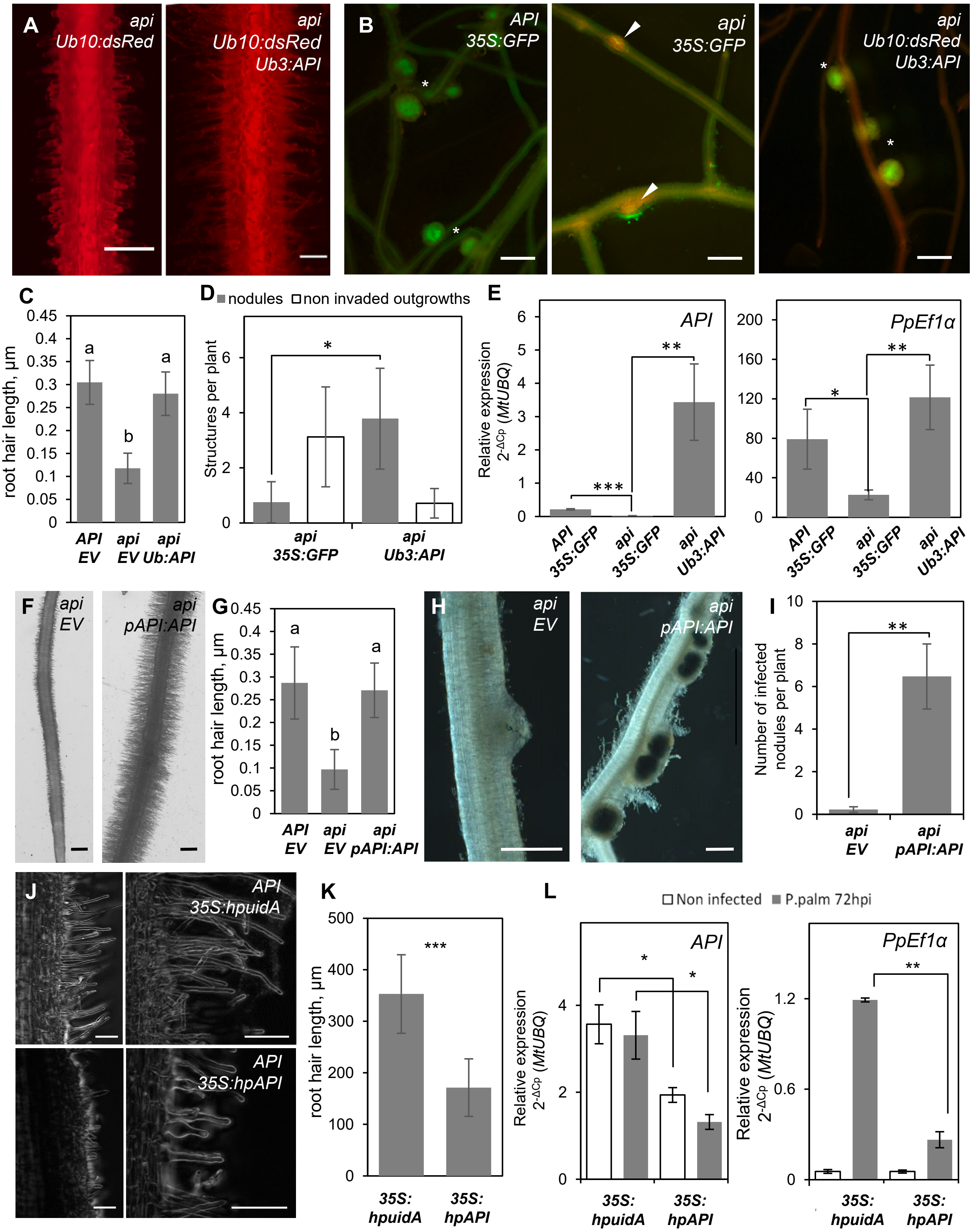

Figure S2. Complementation of *api* mutation phenotypes and knockdown of *API* gene. Related to Figures 1, 2.
Please see next page for the legend.

Figure S2. Complementation of *api* mutation phenotypes and knockdown of *API* gene. Related to Figures 1, 2.
A, Root hair development in *api* hairy roots expressing *Ub10:dsRed* (control) and complemented with *API* gene (*Ub3:API*), scale bars=500µm. **B**, Root nodule development in *API* and *api* hairy roots expressing *35S:GFP* (controls) and complemented with *API* gene (*Ub3:API*) 18 dpi. Asterisks indicate normally developed nodules; arrowheads indicate non invaded outgrowths, scale bars=1mm. **C**, Quantification of *api* root hairs phenotype complementation with *API* gene driven by Ubiquitine promoter *Ub:API* (error bars represent SD; root hairs analysed n=200 per genotype ; One-way ANOVA with post-hoc Tukey HSD Test p<0.05). **D,** Quantification of *api* root nodule phenotype complementation with *API* gene driven by Ubiquitine promoter *Ub:API* (error bars represent SD; plants analysed n=18 per genotype ; t-test: *, p<0.05). **E**, Expression level of *API* gene and *P. palmivora* LILI-YKDel *PpEf1*α biomass marker in *API* and *api* hairy roots expressing *35S:GFP* (controls), and *api* hairy roots expressing *Ub3:API* using the $2^{-\Delta Cp}$ method and *MtUBQ* as a reference gene (error bars represent SD; n=8; t-test: *, p<0.05; **, p<0.01). **F**, Root hair development in *api* hairy roots expressing empty vector (EV) and complemented with *API* gene driven by native promoter (*pAPI:API*), scale bars=500µm. **G**, Quantification of *api* root hairs phenotype complementation with *API* gene driven by native promoter *pAPI:API* (error bars represent SD; root hairs analysed n=200 per genotype ; One-way ANOVA with post-hoc Tukey HSD Test p<0.05). **H,** Root nodule development in *api* hairy roots expressing empty vector (EV) and complemented with *API* gene driven by native promoter (*pAPI:API*) 10 dpi, scale bars=500µm. **I**, Quantification of *api* root nodule phenotype complementation (error bars represent SE; plants analysed n_{API}=18, n_{api}=19; t-test: **, p<0.01). **J**, Silencing of *API* gene caused short root hair phenotype (bars=200µm). **K**, Quantification of *api* root hairs phenotype of *35S:hpAPI* expressing hairy roots (error bars represent SD; t-test: ***, p<0.001). **L,** Expression level of *API* and *P. palmivora* LILI-YKDel *PpEf1*α genes in *35S:hpAPI* and *35S:hpuidA* expressing hairy roots using the $2^{-\Delta Cp}$ method and *MtUBQ* as a reference gene (error bars represent SD; biological replicates n=3; only statistically significant difference is indicated t-test: *, p<0.05; **, p<0.01).

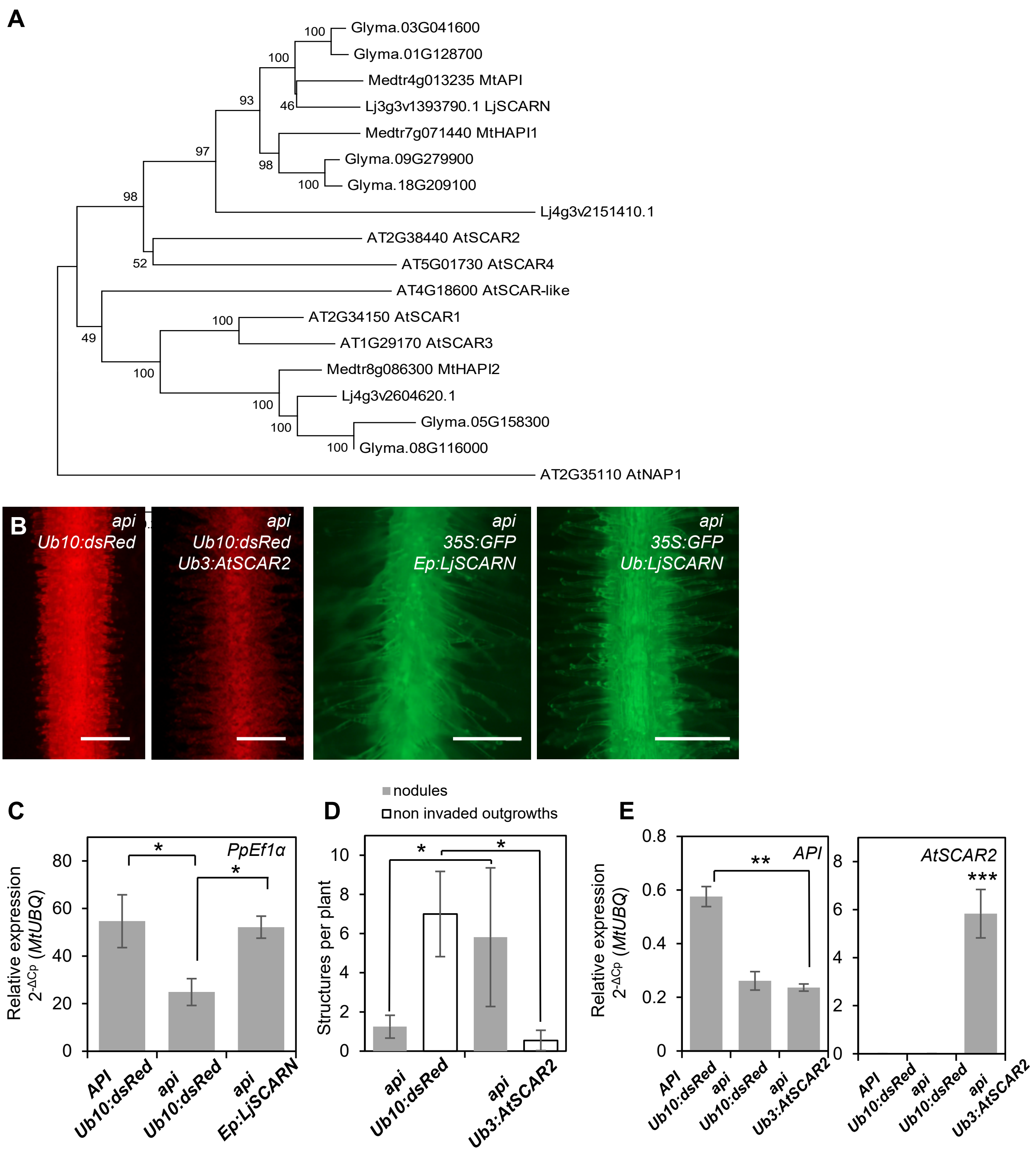

Figure S3. *API* is a member of functionally interchangeable SCAR protein family. Related to Figure 2.
A, The phylogenetic tree of SCAR family proteins were built using MEGA6.06 by using the neighbor-joining method with the bootstrapping value set at 500 replications and Arabidopsis *NAP1* gene as an outgroup. Species: Medtr – *Medicago truncatula*, Glyma – *Glycine max*, Lj - *Lotus japonicus*, AT – *Arabidopsis thaliana*. **B**, Root hair development in *api* hairy roots expressing *Ub10:dsRed* (control) or complemented with Arabidopsis *AtSCAR2* and Lotus *LjSCARN* genes (*Ub3:AtSCAR2; Ep:LjSCARN; Ub:LjSCARN*), bars=500µm. **C**, Expression level of *P. palmivora* LILI-YKDel *PpEf1*α biomass marker in *API* and *api* roots expressing *Ub10:dsRed* (controls), and *api* roots expressing *Ep:LjSCARN* using the $2^{-\Delta Cp}$ method and *MtUBQ* as a reference gene (error bars represent SD; biological replicates n=5; t-test: *, p<0.05; **, p<0.01). **D**, Quantification of *api* root nodule phenotype complementation by expression of *AtSCAR2* gene (error bars represent SD; plants analysed n=15; t-test: *, p<0.05). **E**, Expression level of *API* and *AtSCAR2* genes in *Ub10:dsRed* and *Ub3:AtSCAR2* expressing hairy roots of Medicago using the $2^{-\Delta Cp}$ method and *MtUBQ* as a reference gene (error bars represent SD; plants analysed n=6; t-test: **, p<0.01, ***, p<0.001).

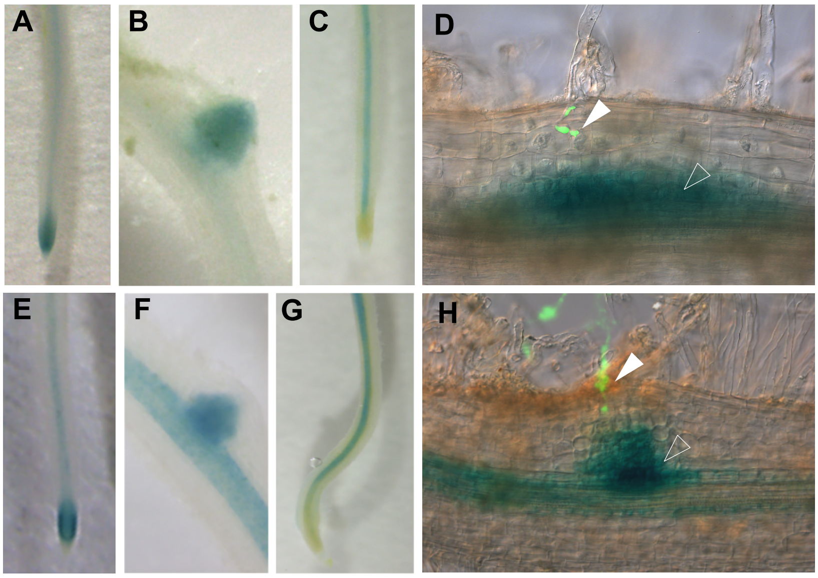

Figure S4. Gene expression analysis of *API* homologs upon microbial colonisation. Related to Figure 3. Expression pattern of *pHAPI1:GUS* fusion in **A,** an apical root meristem and a vascular bundle, **B,** in a lateral root primordium, **C,** in roots at 24hpi with *P. palmivora* AJ-td*,* **D,** in nodule primordia 4 days post inoculation with GFP expressing *S. meliloti.* Expression pattern of *pHAPI2:GUS* fusion in **E,** an apical root meristem and a vascular bundle, **F,** in a lateral root primordium, **G,** in roots at 24hpi with *P. palmivora* AJ-td, **H,** in nodule primordia 4 days post inoculation with GFP expressing *S. meliloti* Open arrowhead indicates dividing cells of a nodule primordia, closed arrowhead indicates an infection thread*.*

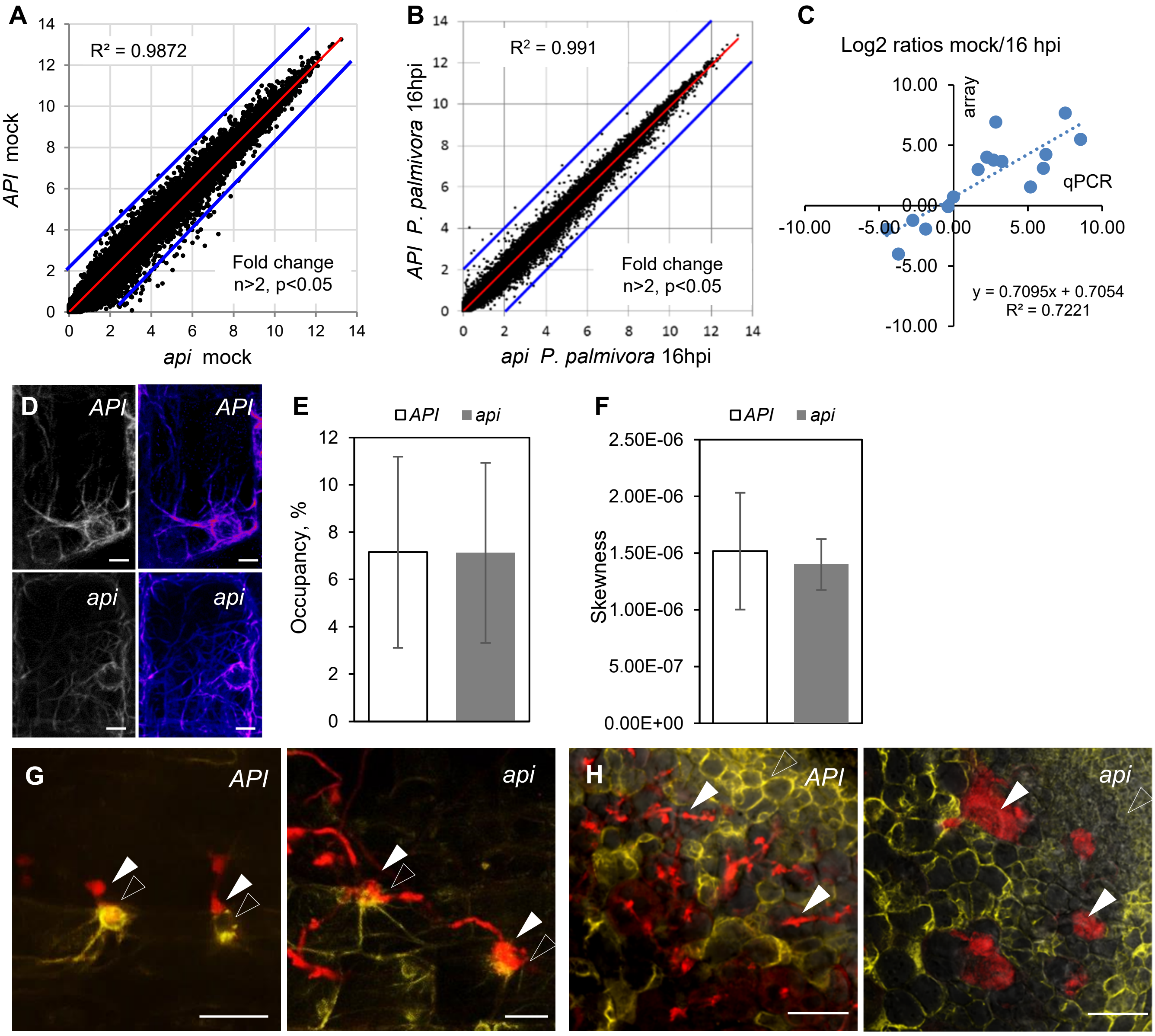

Figure S5. *api* showed unaltered microarray gene expression profiles upon colonisation by *P. palmivora* and a similar extent of actin distribution. Related to Figures 3, 4, Data S1, S2 and STAR methods.

A, Linear regression of log2-transformed absolute expression levels of *M. truncatula* transcripts in mock inoculated *API* and *api* plants. **B**, Linear regression of log2-transformed absolute expression levels of *M. truncatula* transcripts in *P. palmivora* AJ-td inoculated *API* and *api* plants. **C**, qRT-PCR validation of Microarray data at 16hpi with *P. Palmivora* AJ-td using the $2^{-\Delta Cp}$ method and *MtH3l* as a reference gene. Gene expression ratios obtained from microarray and qPCR were plotted as a logarithm (base 2) and correlation between both set of data was calculated. **D-F**, Actin density anlysis: **D**, Projected z-stack of a cortical cell in the elongation zone of *API* and *api* roots expressing a YFP-ABD2 actin reporter (black and white, left column) and skeletonized output after the Fiji 'Analyze Skeleton' plugin application (right column), bars=5µm. **E,** Quantification of actin microfilaments density in *API* and *api* calculated as ratio of the total pixel numbers of actin filaments and cell area (ten transgenic roots were analysed for each genotype; cells analysed: n_{API}=24, n_{api}=22). **F,** Quantification of actin microfilaments bundling in *API* and *api* calculated as the skewness of the intensity distribution of the actin filament pixels (ten transgenic roots were analysed for each genotype; cells analysed: n_{API}=24, n_{api}=22). **G**, *API* and *api* hairy roots expressing YFP-ABD2 actin reporter infected with *P. palmivora* AJ-td 16hpi . Nuclear repositioning (open arrowheads) under the site of appressoria penetration (closed arrowheads) was observed in both *API* and *api* plants (bars=20µm). **H**, *API* and *api* root nodules expressing YFP-ABD2 actin reporter showed no difference in actin distribution in developing root nodules. Open arrowheads indicate nodule meristematic cells, closed arrowhead indicates infections with RFP expressing *S. meliloti* (bars=20µm).

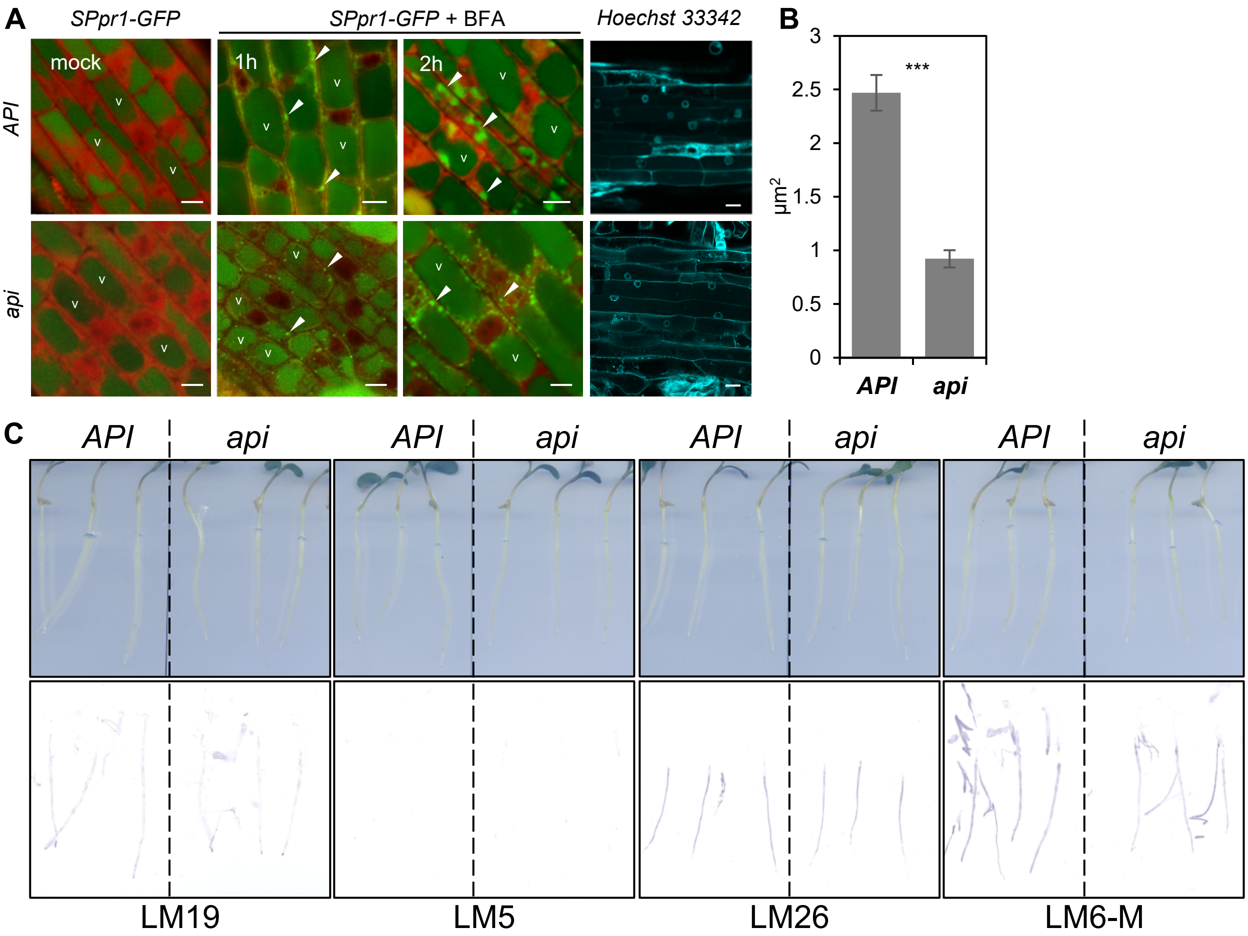

Figure S6. *api* displays impaired endomembrane compartment dynamics and unaltered secretion of homogalacturonan and rhamnogalacturonan. Related to Figure 5.

A, Brefeldin-A induced BFA body formation in *API* and *api* hairy roots expressing secreted GFP after 1-2h treatment with BFA. Arrowheads indicate BFA bodies; v indicates vacuoles. Cytoplasmic red fluorescence originates from *Ub10:dsRed* transgenic root marker expression. Hoechst 33342 staining of *api* and *API* roots showed the same level of dye penetration. A two-hour treatment resulted in staining of 2-3 call layers of *api* and *API* roots. Scale bars represent 10 µm.

B, Quantification of BFA bodies size in *API* and *api* PR1sp-GFP expressing roots after two hours of BFA treatment (error bars represent SE; cells analysed: n_{API}=23, n_{api}=20; t-test: ***, $p<0.001$).

C, Immunodetection of polysaccharides secretion from plant roots. Bright field image of *API* and *api* seedlings grown on agar solid media with the nitrocellulose print of the solid media surface after removal of seedlings which was then probed by monoclonal antibodies specific for homogalacturonan (LM19) and rhamnogalacturonan (LM5, LM26 & LM6-M).

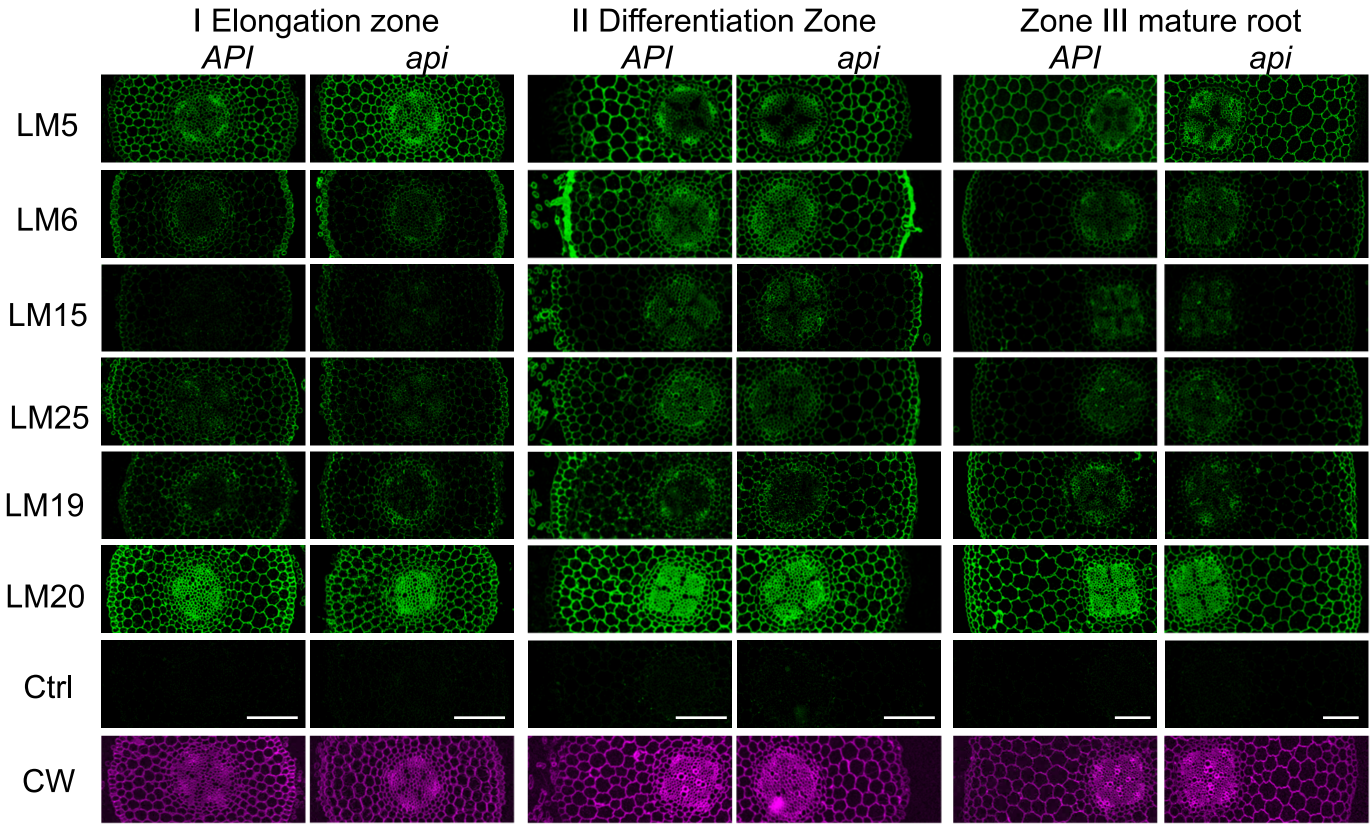

Figure S7. Immunolocalization of different epitopes in *API* and *api* roots at three developmental zones. Related to Figure 6. Ctrl, no primary antibody control**;** CW, Calcofluor White; scale bars=100µm.
